# Effect of food sources of nitrate, polyphenols, L-arginine and L-citrulline on endurance exercise performance: a systematic review and meta-analysis of randomised controlled trials

**DOI:** 10.1186/s12970-021-00472-y

**Published:** 2021-12-29

**Authors:** Noah M. A. d’Unienville, Henry T. Blake, Alison M. Coates, Alison M. Hill, Maximillian J. Nelson, Jonathan D. Buckley

**Affiliations:** 1 Allied Health and Human Performance, University of South Australia, Adelaide, Australia; 2 Alliance for Research in Exercise, Nutrition and Activity (ARENA), University of South Australia, Adelaide, Australia; 3 Clinical and Health Sciences, University of South Australia, Adelaide, Australia

**Keywords:** Nitric oxide, nitrate, polyphenols, l-citrulline, antioxidants, foods, supplements, endurance, athletes

## Abstract

**Background:**

Increasing nitric oxide bioavailability may induce physiological effects that enhance endurance exercise performance. This review sought to evaluate the performance effects of consuming foods containing compounds that may promote nitric oxide bioavailability.

**Methods:**

Scopus, Web of Science, Ovid Medline, EMBASE and SportDiscus were searched, with included studies assessing endurance performance following consumption of foods containing nitrate, L-arginine, L-citrulline or polyphenols. Random effects meta-analysis was conducted, with subgroup analyses performed based on food sources, sex, fitness, performance test type and supplementation protocol (e.g. duration).

**Results:**

One hundred and eighteen studies were included in the meta-analysis, which encompassed 59 polyphenol studies, 56 nitrate studies and three L-citrulline studies. No effect on exercise performance following consumption of foods rich in L-citrulline was identified (SMD=-0.03, p=0.24). Trivial but significant benefits were demonstrated for consumption of nitrate and polyphenol-rich foods (SMD=0.15 and 0.17, respectively, *p*<0.001), including performance in time-trial, time-to-exhaustion and intermittent-type tests, and following both acute and multiple-day supplementation, but no effect of nitrate or polyphenol consumption was found in females. Among nitrate-rich foods, beneficial effects were seen for beetroot, but not red spinach or Swiss chard and rhubarb. For polyphenol-rich foods, benefits were found for grape, (nitrate-depleted) beetroot, French maritime pine, Montmorency cherry and pomegranate, while no significant effects were evident for New Zealand blackcurrant, cocoa, ginseng, green tea or raisins. Considerable heterogeneity between polyphenol studies may reflect food-specific effects or differences in study designs and subject characteristics. Well-trained males (V̇O_2max_ ≥65 ml.kg.min^-1^) exhibited small, significant benefits following polyphenol, but not nitrate consumption.

**Conclusion:**

Foods rich in polyphenols and nitrate provide trivial benefits for endurance exercise performance, although these effects may be food dependent. Highly trained endurance athletes do not appear to benefit from consuming nitrate-rich foods but may benefit from polyphenol consumption. Further research into food sources, dosage and supplementation duration to optimise the ergogenic response to polyphenol consumption is warranted. Further studies should evaluate whether differential sex-based responses to nitrate and polyphenol consumption are attributable to physiological differences or sample size limitations.

**Other:**

The review protocol was registered on the Open Science Framework (https://osf.io/u7nsj) and no funding was provided.

**Supplementary Information:**

The online version contains supplementary material available at 10.1186/s12970-021-00472-y.

## Background

Nitric oxide (NO) is a signalling molecule that is involved in numerous physiological processes including skeletal muscle contraction [1], endothelial function [2], mitochondrial biogenesis and respiration [3], muscle repair [4] and antioxidant defences [5–7]. Given these functions are important during exercise, particularly aerobic exercise, there has been considerable interest in enhancing NO production to improve endurance exercise performance. Specifically, increased synthesis of NO is proposed to reduce the oxygen cost of ATP resynthesis, lower the ATP cost of cross-bridge formation and promote vasodilation, thereby enhancing skeletal muscle blood flow and oxygen perfusion which may speed oxygen uptake kinetics [8]. These effects may translate to increased endurance exercise performance by improving exercise efficiency, decreasing the oxygen deficit at exercise onset and reducing the V̇O_2_ slow component [9].

NO is synthesised through two pathways, one of which is nitric oxide synthase (NOS)-dependent and one which is independent of NOS. NO is synthesised via the NOS-dependent pathway from L-arginine and oxygen in a reaction that is catalysed by various NOS enzymes, including endothelial nitric oxide synthase (eNOS). L-citrulline, an α-amino acid, also contributes to NO synthesis through this NOS-dependent pathway via its conversion to L-arginine. Polyphenols may enhance NO production by increasing eNOS expression and activity [10] and promote NO bioavailability via their antioxidant effects protecting NO from breakdown by reactive oxygen species (ROS). Within the NOS-independent pathway, nitrate (NO_3_^-^) is reduced to nitrite (NO_2_^-^) and then NO. Thus, it is proposed that NO availability may be improved by increasing the availability of NO_3_^-^, NO_2_^-^, L-arginine, L-citrulline, or polyphenols. Such changes in NO bioavailability are typically evaluated by plasma or urine NO_3_^-^ and NO_2_^-^, as these are end products of endogenous NO production, but can also be inferred via measures of vascular function such as flow-mediated dilation (FMD).

In addition to the effects of polyphenols on NO synthesis via the NOS-independent pathway, their antioxidant properties have also been proposed to promote exercise performance by helping to maintain redox balance [11]. While the increase in ROS during exercise is an important signalling component that can facilitate acute responses and chronic adaptations to exercise, an imbalance between oxidative stress and antioxidant capacity may lead to impairment in blood flow, calcium handling and sensitivity, and central neural drive [11–13]. These derangements may contribute to the development of fatigue during exercise, so increasing antioxidant capacity may assist in inhibiting the onset of fatigue and enhance athletic performance. Thus, increased consumption of dietary polyphenols may enhance endurance exercise performance through effects on the NOS-dependent pathway and antioxidant capacity during exercise.

Augmentation of NO synthesis and bioavailability has been attempted by increasing the dietary intake of NO precursors. NO_3_^-^ is abundant in beetroot and green leafy vegetables such as lettuce and spinach [14] and L-arginine is found in seafood, nuts, seeds, soy protein isolate and watermelon. Watermelon is also a rich source of L-citrulline, while fruits, vegetables, tea, coffee and cocoa are rich sources of polyphenols [15]. NO_2_^-^can be found in some fruits and vegetables, with more pronounced concentrations found in processed meats where it is used as an additive [16]. Many foods contain a combination of these nitric oxide precursors, as well as other antioxidant components such as vitamins, minerals and carotenoids, with interactions between these various phytochemicals and food sources potentially resulting in varied responses to specific foods and/or combinations of foods [17–19].

There appear to be considerable inter-individual differences in bioavailability in response to consumption of polyphenols [20] and NO_3_^-^ [21, 22]. Several factors may influence the content and bioavailability of polyphenols in foods, such as storage and processing methods [23], the food matrix, and in particular, the profile of specific polyphenol subclasses (e.g. anthocyanidins vs quercetin) which vary significantly in their absorptive characteristics [20, 24]. Bioavailability is also affected by background diet, genetic factors, and particularly intestinal microbiota, as most polyphenols are catabolised by bacteria in the large intestine and the catabolites then enter the circulation and exert antioxidant effects [19, 25]. Similarly, the composition of NO_3_^-^-rich food sources and microflora of the oral and gut microbiome may impact the bioavailability of NO_3_^-^ from NO_3_^-^-rich foods [22]. While increasing NO production may improve endurance exercise performance, performance effects may also be influenced by sex and fitness-related differences in NO synthesis [26, 27], vascular function [28, 29] and oxidative damage [30, 31]. Thus it is important to evaluate whether the effects of consuming foods that increase NO production on endurance exercise performance are influenced by these individual factors, as well as other parameters such as type of exercise performance test performed, the intensity at which the test is performed, and the dose and duration of consumption of NO precursors.

The primary aim of this systematic review and meta-analysis was to evaluate whether consumption of foods rich in precursors of NO synthesis improves endurance exercise performance. A secondary aim was to determine the effect of dose and duration of consumption of foods rich in NO precursors, participant characteristics (fitness and sex) and exercise test parameters (type and intensity) on exercise performance.

## Methods

### Information sources and search strategy

A literature search was conducted using the Scopus, Web of Science, Ovid Medline, EMBASE and SportDiscus databases on 5 September 2019. Title, abstract, keyword and MeSH (where applicable) searches were implemented.

The search terms are provided in Additional Material 1, but in brief, search terms were grouped under:

1. Population (e.g. human, athlete).
2. Compounds that promote nitric oxide synthesis (e.g. NO_3_^-^, polyphenols) and food sources of these compounds (e.g. beetroot juice, blackcurrant).
3. Terms related to maximal endurance performance (e.g. exercise tolerance, time-trial).

Articles included in the search were exported to Endnote reference management software (Version 9, Clarivate Analytics, Philadelphia, PA), where duplicates were removed before remaining studies were uploaded to Covidence systematic review management software (Veritas Health Innovation, Melbourne, Australia).

### Registration and protocol

A protocol application was submitted to PROSPERO on the 4^th^ of September 2019, but we were informed that it was unsuccessful on the 29^th^ January 2020 as it was not considered within their scope. Thereafter, the authors became aware of the Open Science Framework, where the study was registered on the 5^th^ February 2020 (accessible at https://osf.io/u7nsj), which was after the title and abstract screening had been completed, but before full-text review had been completed by NMAdU or started by HTB.

### Eligibility criteria

Studies were limited to human participants and English language, peer-reviewed studies, with no limit set on publication date. Studies needed to increase dietary intake of compounds that promote nitric oxide synthesis and bioavailability, with such compounds including polyphenols, NO_2_^-^, NO_3_^-^, L-citrulline and L-arginine, and adhere to the inclusion and exclusion criteria listed in Tables 1 and 2 respectively.

**Table 1:** Inclusion criteria

Inclusion criteria	Rationale
Consumption of nitric oxide precursors only via consumption of whole foods, juices, concentrates or plant extracts. Studies must explicitly state the food source(s) of these compounds.	Differences in bioavailability between food and synthetic sources.
Foods could not be consumed in combination with other (non-nitric oxide precursor) foods/supplements that may influence endurance exercise performance (e.g. caffeine) unless the comparator group also contains these components. Such interventions will be included in the qualitative synthesis only.	Confounds the ability to infer the effect of increasing intake of nitric-oxide precursors.
Report an external (or externally derived) measure of maximal endurance exercise performance in a test of at least two minutes durations.	Maximise the contribution of the aerobic energy system to exercise performance and thus provide a measure of endurance exercise performance.
Intermittent-type performance tests required a work duration of at least two minutes and could not have between-repetition recovery intervals of >60 secs	Maximise the contribution of the aerobic energy system to exercise performance and thus provide a measure of endurance exercise performance.
Performance tests conducted in normobaric, normoxic and temperate conditions.	Variant conditions may influence the function of the nitric oxide pathway and the effects of nitric oxide-related supplementation
Where performance tests were acutely repeated (e.g. repeated time trials), only data from the initial performance test were considered eligible.	Performance in subsequent trial(s) may be confounded by initial performance and factors which influence recovery.
Study sample participants aged 18-65 years	Exclude adolescent and older adult populations due to age-based differences in vascular function and oxidative stress

**Table 2:** Exclusion criteria

Exclusion criteria	Rationale
Performance assessed during recreational, open entry events (e.g. marathon)	Lack of controlled conditions
Performance only measured as an indicator of recovery (e.g. hours or days following an exercise stimulus designed to induce damage/stress) rather than being assessed at maximum performance capacity or more closely following a standardised preload	To reduce the influence of post-exercise recovery, which reflects a different mechanism
Treatment comparator was a food rich in nitric oxide precursors (unless differences in content are quantified)	Difficult to infer how much treatment increases nitric oxide precursors relative to control
Supplement contained >1mg caffeine per kg of body mass, as calculated from participant mean mass	Avoid ergogenic effects seen with higher intakes of caffeine [32]
Performance test intensity was acutely regulated by heart rate or oxygen consumption.	Nitric oxide supplements may alter these internal measures and thereby confound the ability to identify effects on endurance exercise performance.

### Study selection

During title and abstract screening, studies were excluded if they were not consistent with the inclusion and/or exclusion criteria. Antioxidant supplements or nutrients from an unspecified source were deemed eligible at this stage, as during preliminary searches it was noted that these types of articles sometimes specified a polyphenol food source from which the supplements or nutrients were obtained in full texts. Full-text articles were then screened to ensure that studies met the eligibility criteria. For inclusion in the meta-analysis, studies needed to report data that would enable calculation of a standardised mean difference (SMD).

Title and abstract screening and full-text review were conducted independently by two reviewers (NMAdU and other authors excluding JDB for title and abstract, NMAdU and HTB for full text), with disagreements regarding eligibility settled by a third reviewer (JDB). Where full texts were unavailable, they were sought from the authors, and reference lists were also pearled to identify additional relevant studies.

### Data collection process and items

The data extraction form was drafted by the lead author (NMAdU), and studies were extracted in duplicate by NMAdU and HTB. Extracted information included date of publication, industry contributions, population, anthropometry, inclusion/exclusion criteria, supplement composition, dosing and duration of supplementation, performance test(s) and outcomes, dietary restriction of other foods or nutrients, and effects on biochemical and physiological markers of nitric oxide production.

### Risk of bias assessment

Risk of bias in individual studies was assessed using the Revised Cochrane risk-of-bias tool for randomized trials [33] by the lead author (NMAdU) and a second author (HTB), with partial duplication to ensure consistency. Risk of bias was evaluated through visual inspection of standard and contoured funnel plots.

### Effect measures

The effects of nitric-oxide related supplements were assessed using endurance exercise performance outcomes. Studies frequently reported multiple performance outcomes from the same performance test (e.g. mean power and time-to-complete reported for a time-trial). Therefore, where applicable, time-to-complete was the extracted outcome used for data analysis from fixed distance/work time-trials, while total distance/work was preferentially extracted for fixed duration time-trials. Likewise, peak power/speed was designated as the primary outcome from graded exercise tests, and total distance/work was extracted as the primary outcome for intermittent tests. Relative mean or peak power outputs were used in favour of absolute values.

Effect size was quantified as the SMD, where the mean difference between treatments was divided by the pooled standard deviation, with Hedge’s correction factor also applied to adjust for small sample sizes [34]. Where performance was assessed before the active and control interventions in parallel trials, SMD was instead calculated from mean change (from baseline) values and standard deviations. Where SD of these changes was unavailable, it was imputed for each group as ${SD}_{change}=\sqrt{SD_{baseline}^2+{SD}_{final}^2-\Big(2\times 0.80\times {SD}_{baseline}\times {SD}_{final}\Big)\kern1em }$ using a correlation coefficient of 0.80 (35, 36). A factor of -1 was applied to SMDs for time to complete to ensure consistent directions of effect against other outcomes. Where separate populations (e.g. male vs female), performance tests or supplementation protocols (e.g. different doses, durations, timings) were implemented within the same study, data were extracted as separate trials. Where standard deviations were not reported, these were calculated using standard error (SE), confidence intervals or *p* values, if available, per the Cochrane Handbook for Systematic Reviews of Interventions [37].

1.1. **Synthesis of results**

Where precise or upper bounds of p-values (e.g. *p*<0.05) of mean differences were provided, standard error (SE) of effect size was calculated using the equivalent *T-*statistic and correlation coefficients between treatment groups were estimated in accordance with Elbourne et al [38]. Where these details were unavailable, SE was calculated in crossover trials as $SE=\frac{1}{N}+\frac{SMD^2}{2N}\times \sqrt{2\left(1-0.72\right)}$, using the lowest correlation coefficient of 0.72 [39]. Effects were defined as trivial (<0.2), small (0.2–0.6), moderate (0.61–1.2), large (1.21–2.0), and very large (>2.0) [40] and precision of the effect size estimate was assessed using 95 % confidence intervals (CIs). Separate meta-analyses were undertaken for crossover and parallel trials.

Data from included studies were qualitatively synthesised. Where suitable data were available, random effects meta-analysis was conducted using STATA/IC software (version 16.1, College Station, TX, StataCorp LLC.) using the inverse variance model and restricted maximum likelihood method. Statistical heterogeneity between subgroups was assessed via Cochran’s Q, while heterogeneity within groups was evaluated by I^2^, with heterogeneity classified as low (I^2^ < 25%), moderate (I^2^ 25–49%) or high (I^2^ > 50%) [41, 42]. Separate meta-analyses were conducted to investigate the effect of study design, blinding, sex, NO precursor (e.g. polyphenols, NO_3_^-^), food source, duration of consumption (i.e. acute vs multiple days), fitness level of study participants, and the mode, type (i.e. time to exhaustion vs time trial) and duration (from time trial and constant load tests only) of exercise performance tests.

For subgroup analyses, fitness was classified by performance level (PL) using the guidelines of De Pauw [43] and Decroix [44] using reported running and cycling maximal oxygen consumption (V̇O_2max_), as provided in Table 3 below. Mixed-sex trials that did not report separate V̇O_2max_ values for females and males, or featured different PLs between the sexes, were excluded from this fitness subgroup analysis. The influence of fitness was also investigated using meta-regression of outcomes against male V̇O_2max_ values.

**Table 3:** Performance Level (PL) Criteria

	PL1	PL2	PL3	PL4	PL5
Female V̇O_2max_ (ml.kg.min^-1^)	<37	37-47.9	48-53.9	52-58	>58
Male V̇O_2max_ (ml.kg.min^-1^)	<45	45-54.9	55-64.9	65-71	>71

## Results

### Study selection and characteristics

**Fig. 1:**
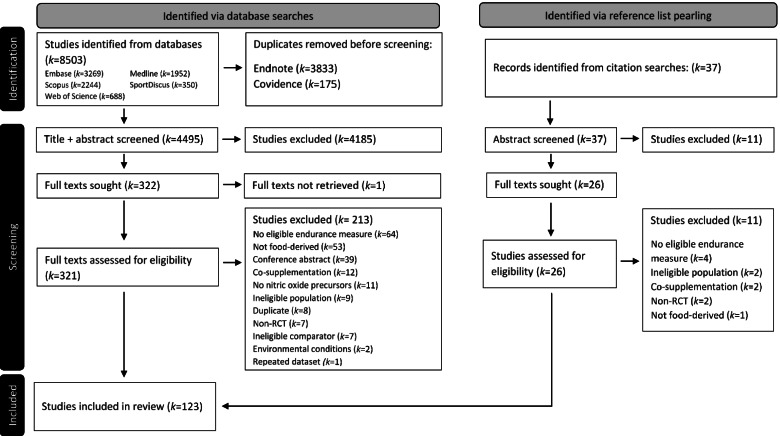
PRISMA flow diagram of search, screening and inclusion outcomes

A PRISMA flow diagram of the search and screening results is displayed in Fig. 1. Of the 123 studies that were eligible for inclusion, 103 used a crossover design and 21 used a parallel design. 101 were reported as double-blind, 13 as single-blind, and 10 did not specify blinding. 62 studies were classified as polyphenol studies, 57 were NO_3_^-^-focused and four studies tested L-citrulline effects, with 40 different food sources represented. While no included studies were specifically focused on L-arginine, it is an additional nutritional component found in watermelon and almonds, and studies that used these foods were classified as L-citrulline and polyphenol studies, respectively. Five studies did not report sufficient data to calculate SMD, thus the meta-analysis incorporated 118 studies. Eleven studies featured multiple performance tests, seven studies assessed the effects of various supplementation durations, five studies evaluated different supplementation dosages, three studies analysed multiple food sources and three studies analysed multiple populations. Thus, the 118 studies with eligible data were considered as 156 separate trials (i.e. with separate effect sizes).

Data from 1872 participants, from 25 different countries were included, comprising 1482 males, 344 females and 46 participants of unspecified sex, as sex was not reported in four studies. From reported study means, pooled mean age was 27.8 ± 5.9 years, with a height of 177.4 ± 10.0 cm, mass of 74.5 ± 9.9 kg and V̇O_2max_ of 53.9 ± 6.9 ml.kg.min^-1^. NO_3_^-^ trials featured a daily dose of 8.4 ± 3.3 mmol NO_3_^-^, polyphenol trials had a daily total phenolic content of 817.4 ± 743.1 mg gallic acid equivalents (where reported), and L-citrulline trials reported a daily dose of 1.71 ± 1.0 g citrulline.

### Risk of Bias

Risk of Bias results for each study (including specific domain of bias scores) are provided in Additional Material 2. Sixty-six studies had a high risk of bias, with 58 studies having ‘some concerns’ and no studies classified at low risk. Classification of high risk was most frequently given for deviations from intended interventions (53%). ‘Some concerns’ was predominant for bias regarding selective reporting of results (100%), reporting of missing outcome data (70%), and the randomisation process (88%).

### Publication Bias

**Fig. 2:**
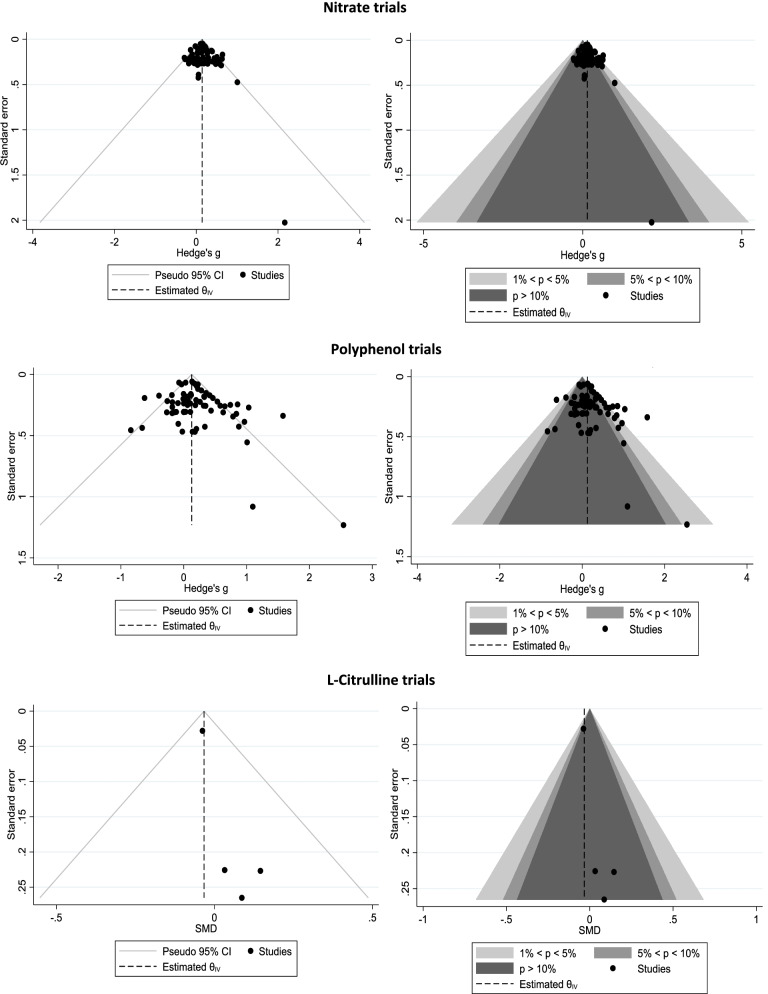
Standard and contoured funnel plots of nitrate, polyphenol, and L-citrulline effects

Standard and contoured funnel plots are displayed in Figure 2. There is some asymmetry within L-citrulline trials that is likely due to the limited number of studies, whereas NO_3_^-^ and polyphenol trials appeared to have good symmetry overall and in the proportion of positive and negative non-significant effects, as assessed by the symmetry within the dark grey shaded areas. Given that 43 trials were designated as receiving financial support, subgroup analyses were also undertaken to help infer whether results from privately funded studies may have been suppressed in the absence of beneficial effects. However, these analyses revealed no significant differences between effects of NO_3_^-^ studies that did or did not receive financial support (p=0.55), whereas financially supported polyphenol studies had significantly smaller effects (p=0.02) than those without private funding. Overall, these results suggest that publication bias did not play a significant role in the meta-analysis results.

### Results of syntheses

#### Nitrate consumption (all food sources combined)

A summary of overall and sub-group analyses is provided in Table 4. Based on the analysis of 81 trials and 956 participants, NO_3_^-^ consumption provided significant, trivial benefits for endurance exercise performance, and trials featured low heterogeneity (I^2^=6.4%). Significant benefits were evident in both crossover (trivial effects) and parallel (small effects) trials, but study design was not a moderator of effects, and results did not differ between study designs (Q_b_=2.37, p=0.12).

**Table 4:** Nitrate meta-analysis and sub-group analyses

	***k***	***n***	**SMD (95% CIs)**	***p***	**I**^**2**^ **(%)**
**Overall**	**81**	**956**	**0.15 [0.12, 0.18]**	**<0.001**	**6.4**
**Design**			*Q*_*b*_*: 2.37*	*0.121*	
Crossover	76	856	0.14 [0.11, 0.17]	<0.001	0
Parallel	5	100	0.36 [0.08, 0.64]	0.011	32
**Blinding**			*Q*_*b*_*: 1.29*	*0.544*	
Single blind	8	77	0.11 [-0.002, 0.22]	0.054	0
Double blind	71	857	0.16 [0.12, 0.19]	<0.001	0
Unclear	2	22	0.07 [-0.14, 0.29]	0.519	60
**Supplementation duration**			*Q*_*b*_*: 3.05*	*0.084*	
Acute	38	437	0.12 [0.06, 0.17]	<0.001	13
Multiple days	43	519	0.18 [0.13, 0.22]	<0.001	9
**Sex**^**1**^			*Q*_*b*_*: 6.94*	*0.031*	
Male	59	658	0.16 [0.12, 0.20]	<0.001	0
Female	4	48	0.0 [-0.12, 0.11]	0.988	0
Mixed	16	229	0.15 [0.09, 0.21]	<0.001	0
**Performance level**			*Q*_*b*_*: 8.78*	*0.032*	
PL1^2^	1	14	-0.29 [-0.69, 0.11]	0.156	-
PL2	23	290	0.24 [0.12, 0.35]	<0.001	52
PL2 females	-				
PL2 males:	17	181	0.24 [0.11, 0.36]	<0.001	52
GXT	3	34	0.16 [-0.14, 0.46]	0.300	80
IGXT	1	14	0.22 [0.01, 0.44]	0.040	
ITTE	1	12	0.47 [0.03, 0.91]	0.038	
TT	4	47	0.01 [-0.28, 0.29]	0.975	53
TTE	8	74	0.39 [0.21, 0.57]	<0.001	27
PL3	15	154	0.18 [0.12, 0.24]	<0.001	0
PL3 females	-				
PL3 males:	15	154	0.18 [0.12, 0.24]	<0.001	0
GXT	2	17	0.1 [-0.27, 0.47]	0.612	53
ITT	2	20	0.17 [0, 0.35]	0.592	0
TT	8	82	0.17 [0.1, 0.24]	<0.001	0
TTE	3	35	0.26 [-0.18, 0.69]	0.249	71
PL4	8	87	0.03 [-0.14, 0.2]	0.762	0
PL4 females	1	14	0.02 [-0.37, 0.42]	0.912	-
PL4 males:	7	73	0.03 [-0.16, 0.22]	0.787	0
GXT	1	12	2.17 [-1.8, 6.14]	0.289	-
TT	6	61	0.02 [-0.17, 0.22]	0.825	0
PL5	6	57	-0.02 [-0.22, 0.17]	0.821	0
PL5 females	1	12	-0.01 [-0.43, 0.42]	0.980	-
PL5 males:	5	45	-0.03 [-0.25, 0.19]	0.808	0
ITTE	1	9	-0.07 [-0.56, 0.42]	0.801	-
TT	4	36	-0.02 [-0.27, 0.23]	0.882	0
**Exercise mode**			*Q*_*b*_*: 2.29*	*0.825*	
Cycling	53	625	0.17 [0.12, 0.22]	<0.001	22
Hand cycling	4	39	0.02 [-0.21, 0.26]	0.859	0
Incline walk/run	1	14	-0.29 [-0.69, 0.11]	0.156	-
Kayaking	2	14	0.13 [-0.02, 0.28]	0.093	0
Rowing	2	20	0.09 [-0.24, 0.42]	0.604	0
Running	15	196	0.14 [0.07, 0.21]	<0.001	19
Swimming	4	48	0.12 [0.004, 0.23]	0.042	0
**Test type**			*Q*_*b*_*: 10.29*	*0.059*	
GXT	15	176	0.15 [0.02, 0.27]	0.023	59
IGXT	2	50	0.13 [0.03, 0.22]	0.008	0
ITT	4	44	0.13 [0.002, 0.27]	0.046	0
ITTE	2	21	0.21 [-0.02, 0.39]	0.244	60
TT	39	436	0.12 [0.07, 0.16]	<0.001	0
TTE	19	229	0.31 [0.20, 0.42]	<0.001	29
**Test duration**			*Q*_*b*_*: 2.89*	*0.577*	
<5 mins:	11	104	0.16 [0.08, 0.23]	<0.001	0
TT	8	70	0.12 [0.04, 0.21]	0.005	0
TTE	3	34	0.32 [0.07, 0.56]	0.011	29
5-10 mins:	24	276	0.18 [0.09, 0.27]	<0.001	31
TT	11	112	0.12 [0.02, 0.22]	0.016	7
TTE	13	164	0.29 [0.13, 0.44]	<0.001	40
10-30 mins:	12	122	0.16 [0.04, 0.28]	0.007	41
TT	9	91	0.12 [-0.004, 0.23]	0.058	41
TTE	3	31	0.44 [0.16, 0.73]	0.002	0
30-60 mins:	5	60	0.08 [-0.11, 0.28]	0.397	0
TT	5	60	0.08 [-0.11, 0.28]	0.397	0
>60 mins:	4	51	0.00 [-0.21, 0.21]	0.986	0
TT	4	51	0.00 [-0.21, 0.21]	0.986	0
Overall (TT & TTE)	56	613	0.15 [0.11, 0.19]	<0.001	2

^1^ Sex not specified in two trials. Abbreviations – GXT, graded exercise test; IGXT, intermittent graded exercise test; ITT, intermittent time-trial; ITTE, intermittent time to exhaustion; k, number of trials; LCI, lower confidence interval; n, pooled sample size; PL, performance level; Q_b_, between-group Q-statistic; SMD, standardised mean difference (Hedge’s g); TT, time-trial; TTE, time to exhaustion; UCI, upper confidence interval

##### Effects by supplementation protocol

Sub-group analyses indicated that trivial benefits existed for both acute and multiple-day supplementation, with no significant difference between the two (p=0.08) and no significant influence of the number of supplementation days (p=0.12). Meta-regression indicated that daily NO_3_^-^dosage of interventions was not associated with performance effects overall (*p*=0.93), or within acute (*p*=0.62) and multiple-day (*p*=0.49) supplementation trials specifically. Sixteen trials restricted intake of other dietary NO_3_^-^ sources, but effects within this subgroup were no different from trials without restrictions (Q_b_=0.55, p=0.46).

##### Sex and fitness-specific effects

Effects differed by classification of participant sex (Q_b_=6.94, p=0.03), as trivial benefits were still seen in male-only and mixed-sex studies, but females demonstrated no effects (SMD=0.0, *p*=0.98) from the results of only four trials. Effects differed by PL overall (Q_b_ = 8.78, *p=*0.03), but when trials were restricted to males only these differences were not significant (Q_b_ = 6.39, *p*=0.09) and meta-regression indicated that V̇O_2max_ was not a significant mediator of the size of performance effects (p=0.10). Most trials included participants classified as PL2, who demonstrated small, significant benefits. Male PL2 (V̇O_2max_: 45-55 ml.kg.min^-1^) trials exhibited a small, significant improvement overall and within TTE trials specifically, but showed no change in TT performance and insignificant trivial benefits for GXT performance. Within the male PL3 bracket (V̇O_2max_: 55-65 ml.kg^-1^.min^-1^), a significant trivial improvement was seen for overall performance, which was still evident for TT performance, while small but insignificant improvements were observed for TTE performance and trivial insignificant effects were indicated for GXT and ITT trials. No effect was present for males in the PL4 (V̇O_2max_: 65-71 ml.kg^-1^.min^-1^) or PL5 brackets (V̇O_2max_ >71 ml.kg^-1^.min^-1^) which were evaluated predominantly through TT performance. Female PL subgroup analyses were not conducted as data were unavailable from female PL2 or PL3 trials and other PLs only included a single study.

##### Effects by test type and duration

Although effects did not vary between performance test subgroups (Q_b_=10.63, p=0.059), non-significant trivial effects were evident for intermittent time trial (ITT) tests, while significant trivial effects were identified for intermittent graded exercise tests (IGXT), time trials (TT) and graded exercise tests (GXT). Small, significant improvements were found in time to exhaustion (TTE) tests, whereas small non-significant benefits were observed for intermittent time to exhaustion (ITTE) tests. There was no heterogeneity within IGXT, ITT or TT trials (I^2^=0%), but moderate heterogeneity was present in TTE (I^2^=29%) trials and high heterogeneity was present for GXT (I^2^=47%) and ITTE (I^2^=60%) trials. Effect sizes within various test duration brackets during TTs and TTE tests are provided in Table 4, and meta-regression found no influence of test duration on performance outcomes for TTs (p = 0.52) or TTE tests (p = 0.48). Since PL2 and PL3 for males were the only categories that demonstrated significant benefits overall, meta-regression was also performed for this subgroup specifically, which found no influence of duration on effects within TTs (*p* = 0.76) or TTE tests (*p* = 0.64).

#### Effects of different nitrate food sources

**Fig. 3:**
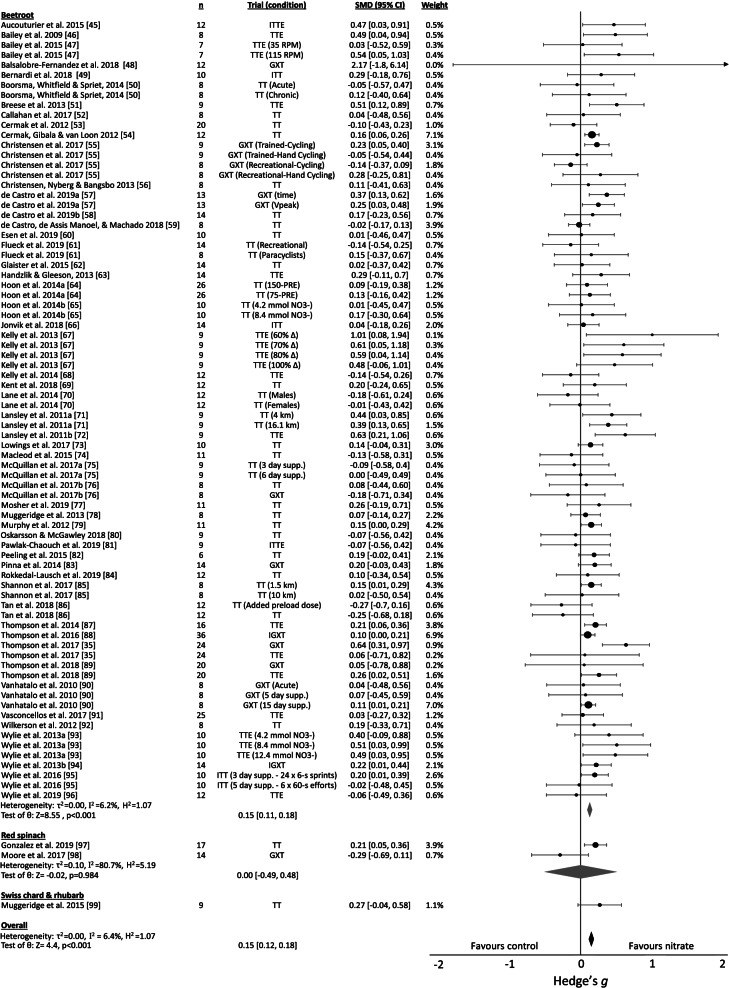
Nitrate supplementation forest plot. Abbreviations – %Δ, work rate that would achieve x% of difference between V̇O_2_ at gas exchange threshold and V̇O_2peak_; CI, confidence interval; GXT, graded exercise test; IGXT, intermittent graded exercise test; ITT, intermittent time-trial; ITTE, intermittent time to exhaustion; k, number of trials; km, kilometre; n, sample size; NO_3_^-^, nitrate; PL, performance level; Q_b_, between-group Q-statistic; rpm, revolutions per minute; s, second; SMD, standardised mean difference (Hedge’s g); supp., supplementation; TT, time-trial; TTE, time to exhaustion.

Sub-group analysis indicated that consumption of (NO_3_^-^-rich) beetroot demonstrated a significant, trivial improvement in exercise performance (Fig. 3), with studies predominantly using beetroot juice as the delivery method (Additional Material 3). No significant effects were evident for consumption of red spinach or Swiss chard and rhubarb from a limited number of studies, but between-food effects were not statistically different between food sources of NO_3_^-^ (Q_b_=1.03, p=0.60).

#### Polyphenol supplementation effects

The analysis of polyphenol effects included 71 trials and 1227 participants, indicating significant, trivial benefits for consumption of polyphenol-rich foods overall (Table 4), with these effects influenced by high heterogeneity (I^2^ = 66%). Forty-five (of 71) trials quantified the polyphenol content of the foods consumed, but the overall effects of these trials were not different from trials where polyphenol content was unspecified, and total phenolic content of the foods consumed did not influence effects (*p*=0.27). There were few differences in subgroup effects (parallel, GXT, TTE, PL1, PL2, and acute trials) in comparison to all polyphenol studies combined (Table 5), and given the similarities between studies that did and did not report polyphenol content, the results presented in-text represent all polyphenol trials. Crossover trials produced trivial, significant benefits, whereas small, insignificant improvements were shown in parallel trials, although subgroup analyses indicated that results between parallel and crossover trials were not significantly different (*p*=0.55).

**Table 5:** Polyphenol meta-analysis and sub-group analyses

	All polyphenol trials					Trials with polyphenols quantified				
	***k***	**n**	**SMD (95% CIs)**	**p**	**I**^**2**^ **(%)**	***k***	**n**	**SMD (95% CIs)**	**p**	**I**^**2**^ **(%)**
**Overall**	**71**	**1227**	**0.17 [0.10, 0.25]**	**<0.001**	**66**	**45**	**829**	**0.20 [0.09, 0.30]**	**<0.001**	**72**
**Design**			*Q*_*b*_*: 0.04*	*0.548*				*Q*_*b*_*: 3.53*	*0.060*	
Crossover	52	730	0.16 [0.08, 0.23]	<0.001	65	34	481	0.14 [0.04, 0.24]	0.006	66
Parallel	19	497	0.24 [-0.02, 0.49]	0.069	56	12	348	0.47 [0.14, 0.81]	0.006	59
**Blinding**			*Q*_*b*_*: 0.34*	*0.845*				*Q*_*b*_*: 0.13*	*0.714*	
Single blind	6	122	0.30 [-0.3, 0.91]	0.329	91	6	122	0.30 [-0.30, 0.91]	0.329	91
Double blind	58	987	0.17 [0.10, 0.24]	<0.001	51	38	674	0.19 [0.08, 0.29]	<0.001	67
Unclear	7	118	0.10 [-0.27, 0.46]	0.621	76	1	33	-0.18 [-0.8, 0.44]	0.572	-
**Supplementation duration**			*Q*_*b*_*: 0.12*	*0.733*				*Q*_*b*_*: 1.46*	*0.228*	
Acute	15	243	0.21 [0.003, 0.41]	0.046	85	5	101	0.56 [-0.07, 1.19]	0.081	95
Multiple days	56	984	0.17 [0.09, 0.25]	<0.001	57	40	728	0.17 [0.07, 0.27]	0.001	61
**Polyphenol quantification**			*Q*_*b*_*: 1.13*	*0.287*						
No	26	398	0.14 [0.04, 0.25]	0.009	52					
Yes	45	829	0.2 [0.09, 0.30]	<0.001	72					
**Sex**^**1**^			*Q*_*b*_*: 7.49*	*0.024*				*Q*_*b*_*: 0.31*	*0.210*	
Male	45	729	0.23 [0.15, 0.32]	<0.001	42	31	556	0.20 [0.08, 0.32]	<0.001	46
Female	4	71	0.01 [-0.19, 0.20]	0.948	0	3	55	0.01 [-0.19, 0.21]	0.942	0
Mixed	19	388	0.05 [-0.09, 0.19]	0.460	78	8	179	0.26 [-0.05, 0.57]	0.098	94
**Performance level**			*Q*_*b*_*: 12.90*	*0.005*				*Q*_*b*_*: 1.17*	*0.558*	
PL1	3	89	0.76 [0.37, 1.15]	<0.001	0	2	80	0.59 [-0.08, 1.25]	0.083	0
PL1 Females	-					-				
PL1 Males:	3	89	0.76 [0.37, 1.15]	<0.001	0	2	80	0.59 [-0.08, 1.25]	0.083	0
GXT	2	80	0.59 [-0.08, 1.25]	0.083	0	2	80	0.59 [-0.08, 1.25]	0.083	0
TT	1	9	0.86 [0.38, 1.34]	<0.001	-	-				
PL2	19	279	0.12 [0, 0.25]	0.059	36	14	207	0.21 [0.09, 0.34]	<0.001	0
PL2 Females	-					-				
PL2 Males:	17	239	0.21 [0.11, 0.32]	<0.001	0	14	207	0.21 [0.09, 0.34]	<0.001	0
GXT	6	94	0.23 [-0.02, 0.47]	0.068	16	6	94	0.23 [-0.02, 0.47]	0.068	16
IGXT	1	26	2.54 [0.13, 4.96]	0.039	-	1	26	2.54 [0.13, 4.96]	0.039	-
ITTE	1	13	0.26 [-0.18, 0.7]	0.252	-	1	13	0.26 [-0.18, 0.7]	0.252	-
TT	6	64	0.18 [0.01, 0.35]	0.042	0	5	54	0.21 [0.02, 0.39]	0.028	0
TTE	3	42	0.21 [0.03, 0.4]	0.021	0	1	20	0.12 [-0.21, 0.45]	0.492	-
PL3	18	275	0.22 [0.08, 0.36]	0.002	31	13	219	0.22 [0.06, 0.34]	0.002	36
PL3 Females	2	44	0.05 [-0.17, 0.28]	0.646	0	2	44	0.05 [-0.17, 0.28]	0.646	0
PL3 Males:	16	231	0.28 [0.14, 0.42]	<0.001	36	11	175	0.28 [0.08, 0.49]	0.006	39
IGXT	1	13	0.54 [0.1, 0.97]	0.016	-	1	13	0.54 [0.1, 0.97]	0.016	-
TT	11	177	0.24 [0.08, 0.41]	0.004	59	9	152	0.24 [0.03, 0.46]	0.029	42
TTE	4	57	0.35 [-0.01, 0.71]	0.056	20	1	10	1.1 [-1.02, 3.22]	0.314	-
PL4	2	19	0.49 [0.21, 0.77]	<0.001	0	1	9	0.41 [0.08, 0.75]	0.015	-
PL4 Females	-					-				
PL4 Males:	2	19	0.49 [0.21, 0.77]	<0.001	0	1	9	0.41 [0.08, 0.75]	0.015	-
TT	2	19	0.49 [0.21, 0.77]	<0.001	0	1	9	0.41 [0.08, 0.75]	0.015	-
PL5	-					-				
**Exercise mode**			*Q*_*b*_*: 6.32*	*0.097*				*Q*_*b*_*: 10.15*	*0.006*	
Cycling	39	589	0.15 [0.06, 0.24]	0.001	54	27	431	0.14 [0.04, 0.23]	0.004	40
Incline Walk/Run	4	83	0.54 [0.21, 0.87]	0.001	0	2	60	0.63 [0.02, 1.24]	0.043	0
Rowing	2	37	-0.32 [-1.34, 0.71]	0.556	63	1	19	-0.84 [-1.74, 0.05]	0.064	-
Running	24	489	0.22 [0.10, 0.33]	<0.001	62	13	290	0.44 [0.17, 0.71]	0.001	82
Swimming	1	11	-0.18 [-0.63, 0.27]	0.434	-	1	11	-0.18 [-0.18, 0.27]	0.112	-
Climbing	1	18	-0.62 [-1.0, -0.25]	0.001	-	1	18	-0.62 [-1.0, -0.25]	0.001	-
**Test type**			*Q*_*b*_*: 10.29*	*0.016*				*Q*_*b*_*: 11.46*	*0.009*	
GXT	17	352	0.14 [-0.001, 0.28]	0.052	23	11	249	0.22 [0.04, 0.39]	0.016	37
IGXT	4	73	1.09 [0.50, 1.68]	<0.001	66	4	73	1.09 [0.50, 1.68]	<0.001	66
ITTE	1	13	0.26 [-0.18, 0.70]	0.252	-	1	13	0.26 [-0.18, 0.70]	0.252	-
TT	35	549	0.12 [0.03, 0.21]	0.011	59	24	370	0.10 [0.01, 0.20]	0.037	41
TTE	14	240	0.16 [0.01, 0.31]	0.037	63	5	124	0.17 [-0.39, 0.72]	0.563	88
**Test duration**			*Q*_*b*_*: 2.40*	*0.663*				*Q*_*b*_*: 5.57*	*0.135*	
<5 mins:	4	49	0.26 [-0.08, 0.59]	0.133	91	1	12	-0.08 [-0.21, 0.06]	0.257	-
TT	2	21	0.36 [-0.55, 1.27]	0.446	93	1	12	-0.08 [-0.21, 0.06]	0.257	-
TTE	2	28	0.20 [0.07, 0.33]	0.002	0	-				
5-10 mins:	9	124	0.08 [-0.14, 0.30]	0.472	73	4	56	-0.11 [-0.43, 0.22]	0.532	76
TT	3	36	-0.01 [-0.16, 0.14]	0.873	0	2	18	-0.02 [-0.17, 0.13]	0.807	0
TTE	6	88	0.11 [-0.23, 0.45]	0.532	80	2	38	-0.25 [-0.97, 0.48]	0.516	88
10-30 mins:	19	339	0.19 [0.12, 0.26]	<0.001	0	12	234	0.22 [0.1, 0.33]	<0.001	14
TT	16	264	0.21 [0.1, 0.32]	<0.001	27	10	176	0.21 [0.06, 0.36]	0.006	35
TTE	3	75	0.18 [0.02, 0.34]	0.031	0	2	58	0.21 [0.03, 0.4]	0.024	0
30-60 mins:	9	122	0.12 [0.03, 0.21]	0.008	0	6	77	0.11 [0.01, 0.2]	0.036	0
TT	7	101	0.12 [0.03, 0.21]	0.008	0	6	77	0.11 [0.01, 0.2]	0.036	0
TTE	2	21	0.04 [-0.32, 0.41]	0.828	19	-				
>60 mins:	5	104	0.14 [-0.38, 0.66]	0.608	83	3	64	0.52 [-0.2, 1.4]	0.206	71
TT	4	76	-0.08 [-0.46, 0.29]	0.673	65	2	36	0.33 [-0.61, 1.27]	0.506	75
TTE	1	28	0.97 [0.21, 1.73]	0.013	-	1	28	0.97 [0.21, 1.73]	0.013	-
Overall (TT & TTE)	46	738	0.14 [0.06, 0.21]	<0.001	59	26	443	0.11 [0.01, 0.21]	0.038	61

^1^ Sex not specified in three trials. Abbreviations – GXT, graded exercise test; IGXT, intermittent graded exercise test; ITTE, intermittent time to exhaustion; k, number of trials; LCI, lower confidence interval; n, pooled sample size; PL, performance level; Qb, between-group Q-statistic; SMD, standardised mean difference (Hedge’s g); TT, time-trial; TTE, time to exhaustion; UCI, upper confidence interval

##### Effects by supplementation protocol

Small, significant benefits were evident for acute supplementation and trivial, significant improvements were exhibited after multiple-day consumption, with high heterogeneity for both acute and multiple-day supplementation. Effects did not differ between acute and multiple-day supplementation (*p*=0.07), nor did the number of supplementation days appear to be a significant moderator of outcomes (*p*=0.47). Twenty-eight of 56 multiple-day studies provided an acute dose before testing, but effects were not different from multiple-day polyphenol trials that did not include a pre-testing dose (*p*=0.37). Seventeen trials restricted intake of foods rich in antioxidants and/or polyphenols, and subgroup analyses indicated no between-group differences with polyphenol trials that had no restrictions (Q_b_=0.74, p=0.39).

##### Effects by sex and fitness

Small, significant benefits were identified for studies with males only, with no effect in female or mixed-sex studies. Small, significant improvements were evident in males in the PL2, PL3 and PL4 brackets, with no eligible studies for PL5, and V̇O_2max_ did not mediate performance effect sizes (*p*=0.76). Low heterogeneity (I^2^=0%) was evident for the male PL2 and PL4 brackets, while moderate heterogeneity (I^2^= 48%) was evident for male PL3 trials. Two female PL3 (V̇O_2max_: 48-52 ml.kg^-1^.min^-1^) trials indicated no effects, with no other female trials available to classify by PL.

##### Effects by duration and test type

Significant, trivial improvements were found for TT and TTE performance, and significant moderate improvements were evident for IGXT, with no significant effects for other test types. One IGXT study [100] likely included a reporting error (very low SD), but even with this trial removed, the effect within IGXTs remained moderate. No significant effects were identified for performance tests with durations or <5 minutes or 5-10 minutes, while trivial, significant benefits were seen in tests lasting 10-30 minutes and 30-60 minutes. Small, but significant performance decrements were seen in performance (all TT) with a duration of >60 minutes. Meta-regression did not indicate a significant influence of exercise duration within TTs (*p*=0.49) or TTE tests (*p*=0.40).

#### Polyphenol-rich foods

**Fig. 4:**
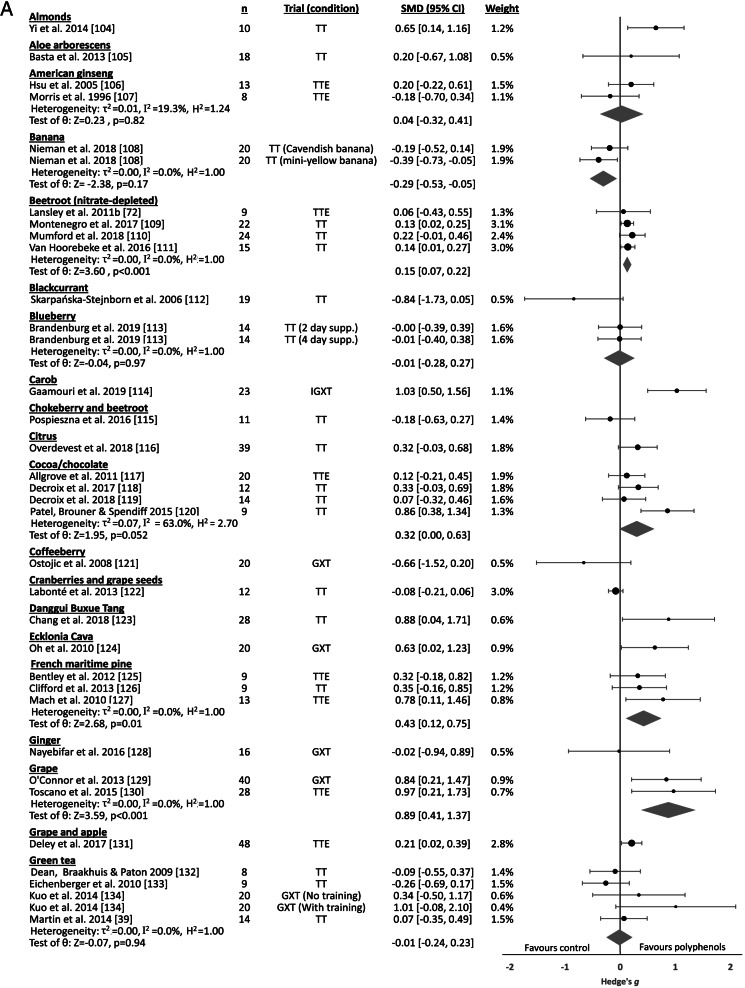

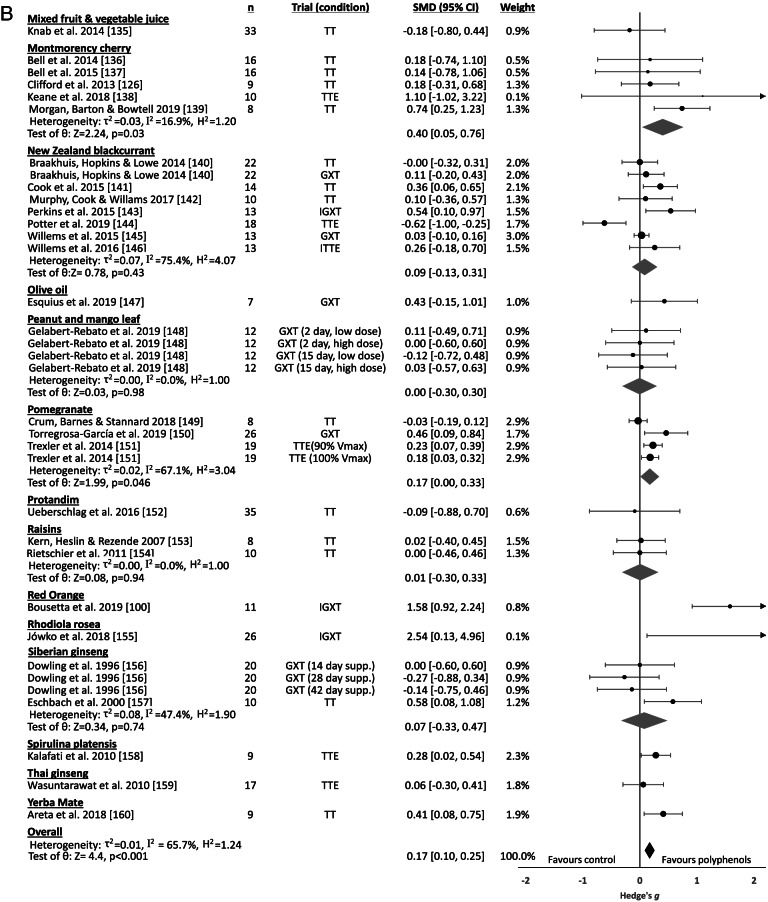
Polyphenol supplementation forest plot**.** Abbreviations – CI, confidence interval; GXT, graded exercise test; IGXT, intermittent graded exercise test; ITTE, intermittent time to exhaustion; k, number of trials; n, sample size; PL, performance level; Qb, between-group Q-statistic; SMD, standardised mean difference (Hedge’s g); supp., supplementation; TT, time-trial; TTE, time to exhaustion; V_max_, maximal running velocity.

Trials evaluating effects of polyphenol-rich foods evaluated 36 separate food sources. Effects on exercise performance are displayed in Fig. 4, with significant differences between food sources (Q_b_=32.64, p=0.002). Moderate, significant benefits were exhibited for the consumption of grape (juice), small significant improvements were seen following consumption of French maritime pine bark extract and Montmorency cherry, and significant, trivial benefits were demonstrated for NO_3_^-^-depleted beetroot and pomegranate. Small effects were found for the consumption of cocoa/chocolate, but this did not reach statistical significance (p=0.052). No effects (SMD<0.1) were evident for the consumption of American or Siberian ginseng, blueberry, green tea, New Zealand blackcurrant, peanut and mango leaf, or raisins, while a significant, trivial performance decrement was evident following banana consumption. Two Panax ginseng studies [101, 102] and one study investigating the effects of honey consumption [103] did not provide sufficient information for inclusion in the meta-analysis, and each of these studies failed to demonstrate significant effects on exercise performance.

#### L-citrulline/Watermelon juice supplementation

**Fig. 5:**
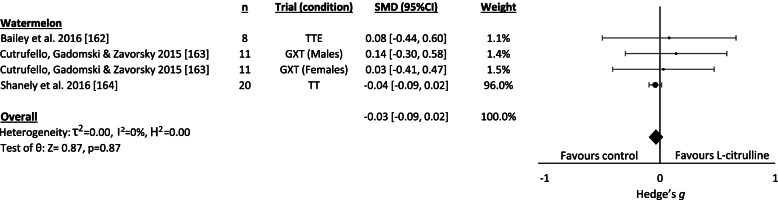
L-citrulline supplementation forest plot. Abbreviations – n, sample size; SMD, standardised mean difference (Hedge’s g); CI, confidence interval; GXT, graded exercise test; IGXT, TT, time-trial; TTE, time to exhaustion; Q_b_, between-group Q-statistic.

L-citrulline (consumed via watermelon juice in all studies) had insignificant trivial detrimental effects on exercise performance based on the results of 4 trials (Figure 5). As shown in Additional Material 4, no significant effects were evident for any subgroup analysis (e.g. exercise mode, type, V̇O_2max_ etc.) and all included studies featured low statistical heterogeneity (I^2^= 0%). An additional study by Tarazona-Diaz et al. [161] not included in the meta-analysis due to insufficient data reporting also failed to demonstrate any effect of watermelon juice on exercise performance.

## Discussion

### Key findings

Consumption of foods rich in NO_3_^-^ and polyphenols exhibited trivial beneficial effects on endurance exercise performance, while no effects of consuming foods rich in L-citrulline were apparent. Grapes, French maritime pine bark and Montmorency cherry, were the most effective food sources of polyphenols, and males may benefit further from polyphenol consumption, with small performance improvements (including small and moderate effects in TT and IGXT performance, respectively). Females do not appear to benefit from consumption of NO_3_^-^or polyphenols, although there are sample size limitations. Fitness does not appear to influence the response to polyphenol consumption, while less-trained male athletes (V̇O_2max_: 45-55 ml.kg.min^-1^) may obtain the greatest benefits from NO_3_^-^ consumption and no effects of NO_3_^-^ are seen in more highly trained athletes (V̇O_2max_: >65 ml.kg.min^-1^).

### Nitrate

#### Findings and proposed mechanisms

This meta-analysis provided evidence that consumption of dietary NO_3_^-^, particularly via consumption of beetroot, provided trivial but significant benefits for endurance exercise performance. This finding corresponds with other reviews that have investigated the ergogenic potential of NO_3_^-^, despite some differences in eligible performance measures (e.g. minimum performance duration, inclusion of non-locomotor performance) and the inclusion of non-food-derived sources such as sodium and potassium NO_3_^-^ [165, 166]. Within this review, NO_3_^-^ consumption displayed significant, small benefits for TTE performance and trivial benefits for TT, GXT performance, with effect sizes aligned with those of McMahon et al. [165]. This review also assessed the effects of NO_3_^-^ on intermittent performance tests, which are more relevant for team-sport athletes given the intermittent nature of their competition, and trivial benefits were found for ITT and IGXT performance. Consistent with Campos et al. [165], we found that effects of NO_3_^-^ on exercise performance are reduced in more highly conditioned athletes. Sub-group analyses indicated that while no effects were evident for athletes with higher V̇O2_max_ values (>65 ml.kg.min^-1^), there were significant small and trivial benefits from NO_3_^-^ consumption in males with a V̇O2_max_ of 45-55 and 55-65 ml.kg.min^-1^, respectively.

Overall, these results suggest that beetroot juice may confer the greatest benefit in males of lower fitness, particularly during TTE tests. The improvements in exercise performance may have resulted from increases in nitric oxide synthesis and vascular function, as inferred via elevated levels of NO_3_^-^and nitrite, and reductions in systolic blood pressure (Additional Material 3), which enhance oxygen delivery and exercise efficiency [8]. These exercise effects were most apparent in less-trained individuals, who demonstrated more frequent reductions in submaximal oxygen consumption and augmentation of tissue oxygenation and oxygen uptake kinetics following NO_3_^-^ supplementation (Additional Material 3). Improvements in exercise performance with NO_3_^-^ intake are believed to be a result of improved mechanical efficiency and V̇O2 kinetics that derive from effects on fast-twitch muscle fibres [8], such as enhancement of excitation-contraction coupling. However, the reduced percentage of fast-twitch fibres and increased expression of calcium handling proteins [167] in elite endurance athletes, may limit these effects, in addition to their increased levels of endogenous nitric oxide synthesis [168, 169], vascular function [26, 29] and habitual dietary NO_3_^-^ intake [170].

#### Food-specific effects of nitrate consumption

While beetroot juice was the predominant food source of NO_3_^-^ in the included trials, which demonstrated trivial benefits for performance, a small number of studies evaluated the effect of NO_3_^-^ from other sources, such as red spinach [97, 98], Swiss chard and rhubarb [99]. These studies reported no benefit for exercise performance; however this may be due to the lower NO_3_^-^ content (~1.5 mmol vs average of 8.4 mmol within included studies) [97, 98] and the assessment of time-trial performance in trained (PL2) males, for which NO_3_^-^ consumption overall demonstrated no benefit [99].

Notably, the included (NO_3_^-^-rich) beetroot studies predominantly used NO_3_^-^-depleted beetroot juice as the comparator, and these studies are therefore assessing the effects of their high NO_3_^-^ content, rather than their overall nutritional properties. Beetroot juice, including commercial concentrated shots (e.g. James White Drinks Ltd, Ipswich, UK) typically used in studies, possesses high total phenolic content and antioxidant activity [171–173], which may confer additional advantages for exercise performance. Lansley et al. [72] found no significant effects of consuming NO_3_^-^-depleted beetroot juice, suggesting that ergogenic effects of beetroot juice are attributable to its NO_3_^-^ content, yet within this review, NO_3_^-^-depleted beetroot supplementation exhibited trivial but significant benefits for performance [72, 109–111]. Thus, the full effects of beetroot may be somewhat underestimated in studies that have used NO_3_^-^-depleted beetroot juice as a comparator.

### Polyphenols

#### Findings and proposed mechanism

The current analysis identified that consumption of polyphenol-rich foods, both acutely and over several days, resulted in trivial but significant effects on endurance exercise performance. This is in agreement with a previous polyphenol meta-analysis [174], although their included interventions included purified polyphenol extracts from unspecified food sources in addition to whole-food extracts. Responses between and within foods varied significantly in the present analysis, as demonstrated by high heterogeneity, which may reflect the complex interplay between the food matrix, its phytochemical and nutritional composition, background diet and other genetic factors that have been shown to produce significant inter-individual variation in the responses to polyphenol consumption [19, 20]. This variation in the response between different foods is typified by both the non-significant influence of total phenolic content on effect size, as well the differential effects between food sources with similar predominant polyphenolic compounds (e.g. anthocyanins in blueberries, cherries and blackcurrant), as can be seen in Table 6 and the phenolic content of interventions listed in Additional Material 3. Further differences in interventions of included studies such as the consumption of single vs multiple polyphenol-rich food sources, the use of whole foods, juices, powders or extracts, as well as restrictions on specific foods or antioxidant sources, may contribute to such heterogeneous responses. Grape, (NO_3_^-^-depleted) beetroot, French maritime pine, pomegranate and Montmorency cherry were the only polyphenol-rich foods that demonstrated significant ergogenic effects, while foods sources such as carob, Danggui Buxue Tang, *Ecklonia cava*, *Rhodiola rosea*, *Spirulina platensis* and yerba mate have shown promising effects within single studies and warrant further investigation.

**Table 6:** Key polyphenols found in review food sources

Group	Subclasses	Food sources	Example compounds
Flavonoids	Anthocyanidins	Berries, cherries, blackcurrants, grapes [15]	Cyanidin
	Flavanols (Flavan-3-ol)	Green tea, cocoa, [15]	Catechin
	Flavanones	Citrus, cherries [15]	Hesperidin
	Flavones	Beetroot, fruit skins, Thai ginseng [15, 175]	Luteolin
	Flavonols	Apples, beetroot, cherries, nuts, olive oil, cranberries [15]	Quercetin
Phenolic acids	Benzoic acid derivatives Cinnamic acid derivatives	Beetroot, nuts, cranberries, ginger, chokeberry [15], Yerba mate [176], ginseng [177–179], cranberries, banana [180], *Aloe arborescens* [181], Danggui Buxue Tang [182], honey [183], Spirulina [184], Protandim [185, 186]	Gallic acid Vanillic acid *p*-coumaric acid Caffeic acid Chlorogenic acid
Stilbenes	Stillbenoids	Grapes, nuts [15]	Resveratrol
Tannins	Hyrdolysable tannins Condensed tannins (Proanthocyanidins) Phlorotannins	Pomegranate, blackcurrant, French maritime bark [187], Carob [188], *Ecklonia cava* [189], *Rhodiola rosea* [190]	Ellagitannins Procyanidin Dieckol

The potential benefits of polyphenols for exercise performance are frequently attributed to their proposed ability to enhance vascular function and limit oxidative damage during exercise by upregulating endogenous antioxidant capacity. However, direct evidence of the physiological mechanisms underpinning the performance changes observed within the included studies was limited. As shown in Additional Material 3, only five polyphenol studies included a direct measure of nitric oxide status (e.g. plasma NO_3_^-^ or NO_2_^-^), with only one study [149] demonstrating a significant increase in any of these biomarkers (plasma NO_3_^-^) following polyphenol (pomegranate) consumption. Similarly, only 14 polyphenol studies investigated effects on vascular parameters such as flow-mediated dilation (FMD) and blood pressure. Improvements in resting FMD were only observed following consumption of cocoa [119] and a cranberry and grape seed extract [122] in well-trained and elite athletes, respectively, while two studies found post-exercise (30-90 minutes) improvements in blood flow [151], and systolic blood pressure [138]. Food-derived polyphenol consumption has had inconsistent effects on vascular function in other healthy populations overall, although consumption of specific polyphenol sources such as tea, cocoa/chocolate and soy has demonstrated more consistent improvements in FMD [191, 192], and thus vascular effects may also be food-specific. However, vascular and skeletal muscle neuronal and endothelial NOS are upregulated by chronic exercise [3], fitness is significantly correlated with nitric oxide production [26, 168] and athletes may also have greater NO bioavailability via enhanced antioxidant defences, all of which may limit the vascular benefits seen in other populations. There was also little evidence for other mechanisms of action, such as improvements in markers of oxidative stress and muscle damage, or internal performance parameters (e.g. oxygen consumption, blood lactate accumulation). Thus, the mechanisms by which polyphenol-rich foods improve endurance exercise performance requires further consideration.

##### Influence of fitness

While the positive effects of NO_3_^-^ consumption appeared to decrease with greater levels of aerobic fitness, such a relationship was not apparent in polyphenol trials, as small benefits were evident across a range of fitness levels (PL1, PL3 and PL4). When restricted to males only, small, significant benefits were shown in PL2 trials. Of considerable interest is that within males, small effects remained for TT performance within each PL3 & PL4 (only one TT trial in PL1). A 2017 meta-analysis of the performance effects of polyphenols, though not restricted to food sources, also reported that training status did not affect response to supplementation, with polyphenols having a significant ergogenic effect overall [174]. Unlike NO_3_^-^ trials, no data were available for PL5 athletes within this review, although several studies have been conducted on high-level endurance athletes where V̇O2_max_ data was unavailable, or a PL was not allocated due to test modality differences. No effects were seen following polyphenol supplementation in national-level rowers [105, 112], a cohort of elite athletes (primarily speed-skaters) [122], or national-level runners [147], although the latter two studies [122, 147] utilised a performance test mode that was not specific to their discipline. Given the lack of polyphenol studies evaluating the responses of elite endurance athletes, the efficacy of polyphenol consumption for this population remains unclear and warrants further investigation.

##### Influence of polyphenol consumption protocol (dosage & supplementation duration)

Several sub-group analyses were used to investigate the possible influences of dose and duration of consumption on the response to polyphenol-rich foods. Regression analysis indicated that total phenolic content was not a significant moderator of effects, suggesting a lack of a dose-response relationship between polyphenol intake and endurance exercise performance. Further, two included studies provided separate effect sizes for responses to differing levels of polyphenol content of the same food, and while these studies indicated no dose-response relationship, neither study induced a significant effect in response to any dosage [108, 148]. There were also no significant differences in the effects between acute and multiple-day consumption, suggesting no influence of duration of consumption. Three studies investigated the responses to polyphenol consumption across different supplementation periods but showed no effect for any supplementation length [113, 148, 156]. Also, while some multiple-day NO_3_^-^ loading protocols included an additional acute dose approximately 2.5 hours before exercise performance testing, only half of the multiple-day polyphenol trials reported the use of an acute dose (see Additional Material 3). Pharmacokinetic analyses have indicated that plasma polyphenol concentrations typically peak around 2.2 hours post-ingestion [193], yet effects between multiple-day polyphenol trials that did or did not include an additional acute dose before performance testing were equivocal. Given the unclear role of food total phenolic content, variation in pre-exercise consumption timing and similar results between acute and multiple-day supplementation, these results do not provide clear insights into recommendations for consumption protocols that can optimise the ergogenic response to polyphenol-rich foods.

### L-Citrulline/Watermelon Juice

Four watermelon juice studies were included within the review, which were conducted in participants of multiple fitness levels (two trials each for PL2 and PL3) and assessed performance through GXTs, ITTs, TTs and TTE tests. However, the included studies had significant differences in the l-citrulline content of the watermelon juice used. Bailey et al. [162] used concentrated watermelon juice containing 2.3-3.4 times the l-citrulline content in the pureed juice used in other included studies [161, 163, 164] and was the also only study to demonstrate an increase in pre-exercise plasma NO_3_^-^ or any marker of nitric oxide synthesis (Additional Material 3). Despite increases in submaximal tissue oxygenation, Bailey et al. [162] indicated minimal effects on exercise performance, oxygen consumption or oxygen uptake kinetics, and no other studies noted improvements in internal performance indicators. Thus, no effect of watermelon juice consumption on exercise performance was observed overall, or within any individual study.

### Areas for future research

#### Sex differences in effects of nitric oxide-related supplementation

While trivial effects were evident for the consumption of NO_3_^-^ and polyphenol-rich foods, neither nutrient was effective in enhancing female endurance exercise performance (g=0.0 and 0.01, respectively) based on data from four trials. Polyphenol mixed-sex trials also demonstrated no effect on exercise performance, whereas trials of males only demonstrated small, significant effects. This finding is in agreement with that of a polyphenol meta-analysis by Somerville, Bringans and Braakhuis [174], who noted an attenuated effect (magnitude not specified) of exercise performance in studies that included both females and males.

Regarding the lack of efficacy of NO_3_^-^ consumption for improving exercise performance in females, it is difficult to determine whether the lack of effect is attributable to biological differences between the sexes or the contexts of the studies. All four trials that assessed the effect of NO_3_^-^ on exercise performance in females assessed time-trial performance, and were predominantly conducted in high-level athletes, with two studies conducted in participants classified as PL4 [62] and PL5 [70], and one study conducted in national-level water polo players. Hence, the absence of effects in these trials may be attributable to the decreased efficacy demonstrated for both time-trial performance and fitter athletes within this review, although an included study by de Castro, de Assis Manoel, and Machado [59] found no effects in untrained females. Overall, the small number of female trials, particularly in less well-trained athletes, combined with the similar effect sizes between mixed-sex (although these were predominantly comprised of males) trials and male-only trials, makes it difficult to infer whether there are genuine sex differences in the response to NO_3_^-^ consumption.

If sex differences in the response to polyphenol and NO_3_^-^ consumption do exist, several potential mechanisms may be responsible. Females have increased endothelium-dependent dilation [28], which may be attributable to elevated levels of plasma nitrite as a result of enhanced NO_3_^-^-reducing activity by oral bacteria [194] and an augmenting role of estrogen in eNOS expression [27]. The increased proportion of slow-twitch muscle fibres in females [195] may be an additional factor, given that NO_3_^-^ consumption proposedly confers its ergogenic potential primarily via effects on fast-twitch muscle fibres [8]. Females also appear to have reduced levels of oxidative stress compared with males, which may be due to a variety of factors including the antioxidant properties of estrogen [30]. While these factors may provide mechanistic underpinnings for the reduced response observed in females, studies in females are underrepresented in the literature, comprising only 18% of the participants within the meta-analysis and female-only studies were even more limited. Thus, further research of potential sex differences in the response to NO_3_^-^ and polyphenol consumption is certainly still warranted.

#### Effects of exercise intensity on responses to supplementation

Metabolic acidosis is proposed to inhibit NO synthesis through the NOS-dependent pathway while enhancing synthesis through the NOS-independent pathway [196], which would suggest distinct intensity-dependent effects of NO_3_^-^ and polyphenol consumption. However, there appeared to be no moderating influence of test duration on effect sizes for TT or TTE tests following both NO_3_^-^ and polyphenol consumption. Several studies assessed the effects of NO_3_^-^ and polyphenol consumption across multiple exercise intensities, with mixed results. Investigating the effects of NO_3_^-^ on TTE at increasing exercise intensities, Kelly et al. [67] reported decreasing effect sizes (Hedge’s *g* = 1.06, 0.64, 0.62 and 0.48, respectively) as intensity increased and no significant effect at maximal intensity (V̇O_2peak_). Similarly, [151] Trexler et al. found significant TTE improvements at 90% and 100%, but not 110%, of peak aerobic velocity following pomegranate extract consumption. Two studies conducted multiple distance TTs following NO_3_^-^ consumption, with Shannon et al. [85] noting positive effects in a 1.5 km but not 10 km running TT, whereas no differences between improvements were seen between 4 and 16.1 km cycling TT performance by Lansley et al. [71]. Regarding intermittent performance, Wylie et al. [95] found significant increases in mean power output during 24 repeated six-second sprints, but no effect on exercise performance across six 60-second efforts. Overall, these studies do not provide a clear indication of whether the effectiveness of consuming foods that promote synthesis of nitric oxide may be influenced by the intensity/duration of the performance test.

#### Potential influence of other dietary factors

The bioavailability of polyphenols is dependent on several factors including the phytochemical and overall composition of their food source matrix, background diet and genetic factors, particularly intestinal microbiota [25, 191]. Most included studies increased polyphenol intake through supplementation with a specific product or food, rather than a more holistic dietary intervention that increases polyphenol intake from various food sources. Notable exceptions included Knab et al. (2014), who utilised a fruit and vegetable juice blend powder, while a study by Ueberschlag et al. (2016) used Protandim, a mix of milk thistle, bacopa, ashwagandha root, turmeric and green tea, but no significant performance improvements were seen in either of these studies. There is evidence that interactions between specific combinations of polyphenols and their food sources can have both synergistic and inhibitory effects on antioxidant activity [18, 197], but whether this may influence exercise performance is not yet established. It has also been postulated that the co-ingestion of NO_3_^-^ and polyphenols could have a synergistic effect on nitric oxide status [18, 191], which may contribute to increased efficacy of NO_3_^-^ administered as beetroot juice in comparison to sodium NO_3_^-^ [198], although limited evidence is available in support of this [17]. Baker et al. [199] found significant improvements in TT performance following a four-day Mediterranean diet intervention where consumption of olive oil, fruits, nuts, seeds and vegetables was significantly increased, although there were also changes in intake of fish and red meat. Thus, while food-derived polyphenol consumption was shown to have significant overall benefits within this review, further research is still warranted into whether polyphenol consumption through a more holistic, whole foods-based approach may still enhance endurance exercise performance and whether the co-consumption of polyphenols NO_3_^-^-rich foods could confer any additional ergogenic effects.

Given the potential interactions of NO_3_^-^ and polyphenols with other dietary factors, it is also of interest as to whether nitric oxide-related supplements may affect the responses to other ergogenic aids such as caffeine. Four beetroot studies investigated the effects of beetroot juice both alone and in combination but indicated that beetroot juice had neither a positive effect independently, nor did it influence the ergogenic effect of caffeine [62, 63, 70, 80]. These studies were all conducted on highly-trained athletes (all ≥PL4) and three of the four studies assessed performance via a TT, with both factors having reduced ergogenic effects within this review. No included polyphenol studies investigated any interactional effects with caffeine, although an excluded study investigating the effects of consuming coffee rich in caffeine and chlorogenic acid demonstrated no effects on TT performance despite comparison against a decaffeinated placebo [200].

#### Effects on training adaptations

While it appears that NO_3_^-^ and polyphenol consumption can enhance endurance exercise performance, it is also important to evaluate whether their consumption may influence an athlete’s response to training. While most studies controlled each individual’s training performed during the interventions, several studies investigated responses to polyphenol consumption during a training program. NO_3_^-^-rich beetroot juice consumption enhanced adaptions to sprint interval training [35, 89], which may be related to enhancing exercise capacity during training [201], as well as remodelling of skeletal muscle towards oxidative phenotypes [35], although this has not been consistently found [89]. While Kuo et al. [134] found no effects of green tea consumption on its own, an ergogenic response was shown when it was combined with an endurance training protocol. This interactional effect has not been replicated by other polyphenols studies however, with no discernible effects evident following consumption of ginger and New Zealand blackcurrant in combination with high-intensity interval training [128, 140], although both of these studies were in females. It has been proposed that there is a hormetic relationship between the oxidative stress induced by training and subsequent adaptations, whereby an optimal amount of oxidative damage is needed to maximise training adaptations, whereas inadequate or excess levels can both result in negative responses [202]. Indeed, chronic antioxidant supplementation with vitamin E has been linked to impaired performance [203], and varied responses have been demonstrated following vitamin C supplementation [204]. Presently, the lack of negative effects overall and in response to the same training suggests that consumption of polyphenol and NO_3_^-^-rich foods does not impair adaptations to training, although their ability to augment these adaptations requires further investigation.

## Limitations

### Polyphenol inclusion criteria

One limitation of identifying polyphenol-rich foods within the present study was that there is no set definition of what this entails, and often studies did not specify phenolic content or reported only the composition of specific polyphenolic compounds and total phenolic content was unclear. This issue was identified during the development of the search strategy, and thus it was decided that a separate meta-analysis would also be conducted for studies that did report phenolic content. However, as seen in Table 6, results largely did not differ between studies that did and did not report phenolic content.

Several foods featured in the included studies also contained other bioactive compounds (e.g. other antioxidants such as polysaccharides, carotenoids, ginsenosides, vitamins C and E) that may confound the effects of their phenolic content. While there are some exceptions [184], in many such foods, antioxidant activity remains very strongly linked to total phenolic content [178, 205–207], suggesting that polyphenols are the primary driver of their antioxidant properties. Also, in the interest of evaluating the effectiveness of these foods overall and maintaining ecological validity, foods were deemed eligible unless such compounds were added to foods separately.

### Risk of Bias

In contrast to older reviews that used the previous Cochrane RoB Tool, no studies within this review were classified as low risk using the RoB Tool 2.0, reflecting its more explicit, detailed requirements that were not aligned well with sports nutrition and exercise science reporting standards. Future publications should provide more specific details to better ascertain risk, particularly regarding specification of the randomisation process used, allocation sequence concealment, compliance with dietary interventions, and reference to pre-specified data analysis protocols, as these were poorly addressed by included studies.

## Conclusion

Consumption of foods rich in NO_3_^-^ and polyphenols may provide trivial beneficial effects for endurance exercise performance, while consumption of foods rich in L-citrulline, currently limited only to studies of watermelon juice, does not appear to affect performance. Beetroot juice has been extensively studied and its NO_3_^-^ content confers ergogenic effects in various exercise types in populations that are not considered well-trained. Other food sources of NO_3_^-^ require further investigation of their ergogenic capacity. Food-derived polyphenols appear to have the potential to enhance TT performance to a similar extent as beetroot juice, although more research is needed regarding its efficacy for use in highly trained athletes. No effects were evident for the consumption of polyphenols from New Zealand blackcurrant, cocoa, ginseng, green tea and raisins, but significant benefits were shown for the consumption of grape, beetroot (NO_3_^-^-depleted), French maritime pine, Montmorency cherry and pomegranate across multiple studies. However, caution should be exercised in translating these ergogenic effects to other food sources of polyphenols, as there seems to be considerable variation in the effects between foods that cannot be attributed to differences in total phenolic content or key polyphenolic compounds. Distinct responses to NO_3_^-^ and polyphenol supplementation were also observed between males and females, with females not demonstrating any benefit for exercise performance. This may be due to sex-based differences in nitric oxide synthesis, vascular function and oxidative stress, and/or the limited number of female studies and the training status of the participants. NO_3_^-^-rich food consumption increases nitric oxide synthesis, and its physiological effects are more clearly linked to increases in muscle oxygen delivery and exercise efficiency, whereas polyphenol-rich foods have less clearly established effects on nitric oxide synthesis, vascular function and physiological variables during exercise. Future studies should evaluate effects of NO_3_^-^ and polyphenol consumption on training performance and adaptations, as well as optimising protocols for consuming polyphenol-rich foods, and establishing the individual and test-related (e.g. intensity) factors that influence the ergogenic response to consuming NO_3_^-^ and polyphenol-rich foods.

## Supplementary Information

**Additional file 1.** Database search strategies. Verbatim search strategy used in each database.

**Additional file 2. **Cochrane Risk of Bias Tool 2.0 Summary. Assessments of overall and domain-specific bias of included studies.

**Additional file 3.** Study Characteristics and Summary Table. Description: Key characteristics and results of included studies.

**Additional file 4. **L-citrulline meta-analysis and sub-group analyses. Description: L-citrulline meta-analysis and sub-group analyses table.

## Data Availability

The datasets used and/or analysed during the current study are available from the corresponding author on reasonable request.

## Abbreviations

GXT: graded exercise test
IGXT: intermittent graded exercise test
ITT: intermittent time-trial
ITTE: intermittent time to exhaustion
k: number of trials
km: kilometre
n: sample size
NO_2_^-^: nitrite
NO_3_^-^: nitrate
PL: performance level
Q_b_: between-group Q-statistic
SMD: standardised mean difference (Hedge’s g)
TT: time-trial
TTE: time to exhaustion
