# Supplementary material for: Effect of food sources of nitrate, polyphenols, L-arginine and L-citrulline on endurance exercise performance: a systematic review and meta-analysis of randomised controlled trials
Source: J Int Soc Sports Nutr. 2021 Dec 29;18:76. doi: 10.1186/s12970-021-00472-y (PMC8715640; doi:10.1186/s12970-021-00472-y)
Supplement: Supplementary file 1 — Additional file 1. Database search strategies. Verbatim search strategy used in each database. [file 12970_2021_472_MOESM1_ESM.docx]

Noah MA d’Unienville ^a,b^_,_ Henry T Blake ^a,b^_,_ Alison M Coates ^a,b^_,_ Alison M Hill ^b,c^_,_ Maximillian J Nelson ^a,b^ & Jonathan D Buckley ^a,b^_,_  ‘Effect of food sources of nitrate, polyphenols, L-arginine and L-citrulline on endurance exercise performance: a systematic review and meta-analysis of randomised controlled trials’_,_ *Journal of the International Society of Sports Nutrition*

^a^ Allied Health and Human Performance, University of South Australia, Adelaide, Australia

^b^ Alliance for Research in Exercise, Nutrition and Activity (ARENA), University of South Australia, Adelaide, Australia

^c^ Clinical and Health Sciences, University of South Australia, Adelaide, Australia

Corresponding Author: Noah M. A. d’Unienville - Contact email: Noah.D'Unienville@unisa.edu.au

**Online Resource 1:** Database search strategies

**Ovid Medline Search Strategy**

Humans/ or Athletes/ or human? or athlete? or runner? or swimmer? or rower? or cyclist? AND Nitric Oxide/ or Nitrates/ or Nitrites/ or Polyphenols/ or Flavonols/ or Flavonoids/ or Anthocyanins/ or Catechin/ or Nitric Oxide Synthase Type III/ or Citrulline/ or Arginine/ or Antioxidants/ or Beta vulgaris/ or Cacao/ or Chocolate/ or Citrullus/ or Grape Seed Extract/ or Punicaceae/ or Prunus avium/ or Ribes/ or Spinacia oleracea/ or Tea/ or Vaccinium macrocarpon/ or Vitis/ or "Nitric Oxide" or "Nitric acid derivative" or Nitrate* or Nitrite* or Polyphenol* or Flavonol* or "Flavanol derivative" or Flavonoid* or Anthocyanin* or Catechin* or "Endothelial Nitric Oxide Synthase" or ENOS or Citrulline or Arginine or Antioxidant* OR beta vulgaris or beet or beetroot* or blackcurrant* or Ribes or blueberr* or cranberr* or Vaccinium macrocarpon or berry or grape* or pomegranate or Punica* or cacao or cocoa or chocolate* or dark chocolate* or spinach* or red spinach* or watermelon* or Citrullus* or cherr* or Prunus avium or black tea* or green tea* AND Exercise Tolerance/ or Cardiorespiratory fitness/ or Physical endurance/ or Running/ or Bicycling/ or

Endurance Training/ or Swimming/ or "Exercise Tolerance" or "Cardiorespiratory fitness" or "Physical endurance" or "Endurance Training" or Exhaustion or "endurance performance" or "time-trial performance" or "time trial performance" or "cycling adj3 performance" or "running adj3 performance" or "swimming adj3 performance" or "rowing adj3 performance" or "time adj3 fatigue" or "aerobic power" or "aerobic capacity" or "aerobic performance" or "maximal oxygen uptake" or "intermittent exercise" or "repeated sprint" or "high?intensity exercise" or "Yo-Yo Intermittent Recovery Test" or "high intensity exercise" or "shuttle test"

**EMBASE Search Strategy**

cyclist/ or runner/ or marathon runner/ or human/ or athlete/ or human? or athlete? or runner? or swimmer? or rower? or cyclist? AND Nitric Oxide/ or Nitric acid derivative/ or Nitrite/ or Polyphenol/ or Flavanol derivative/ or Flavonoid/ or Anthocyanin/ or Nitric Oxide Synthase Type III/ or Citrulline/ or Arginine/ or Antioxidant/ or Blackcurrant/ or Black tea extract/ or Beetroot/ or Beetroot Juice/ or Berry/ or Blueberry/ or Blueberry extract/ or Cacao/ or Cherry/ or Chocolate/ or Cranberry/ or Cranberry juice/ or Cranberry extract/ or Grape/ or Grape Juice/ or Grape seed extract/ or Pomegranate/ or Pomegranate extract/ or Pomegranate juice/ or Ribes/ or Spinach/ or Sweet cherry/ or Tea/ or Vitis/ or Watermelon/ or Nitric Oxide" or "Nitric acid derivative" or Nitrate* or Nitrite* or Polyphenol* or Flavonol* or "Flavanol derivative" or Flavonoid* or Anthocyanin* or Catechin* or "Endothelial Nitric Oxide Synthase" or ENOS or Citrulline or Arginine or Antioxidant* or (beta vulgaris or beetroot* or blackcurrant* or Ribes or blueberr* or cranberr* or Vaccinium macrocarpon or berry or grape* or vitis or pomegranate* or Punica* or cacao or cocoa or chocolate* or dark chocolate* or spinach* or Spinacia oleracea or red spinach* or watermelon* or Citrullus* or cherr* or Prunus avium or tea or black tea* or green tea* AND Exercise tolerance/ or Cardiorespiratory fitness/ or Cardiopulmonary exercise test/ or Endurance/ or Running/ or Cycling/ or Swimming/ or Swimming speed/ or Endurance training/ or Rowing/ or Aerobic capacity/ or Exhaustion/ or Exercise Tolerance or Cardiorespiratory fitness or Physical endurance or Endurance Training or Cardiopulmonary exercise test or endurance performance or Exhaustion or Time-trial performance or cycling) adj3 performance) or running) adj3 performance) or swimming) adj3 performance) or rowing) adj3 performance) or time) adj3 fatigue) or time trial performance or aerobic power or aerobic capacity or aerobic performance or maximal oxygen uptake or intermittent exercise or repeated sprint or high-intensity exercise or high intensity exercise or Yo-Yo Intermittent Recovery Test or shuttle test

**Web of Science Search Strategy**

(TS=(human? OR athlete? OR runner? OR swimmer? OR rower? OR cyclist?) **AND** TS=("Nitric Oxide" or "Nitric acid derivative" or Nitrate* or Nitrite* or Polyphenol* or Flavonol* or "Flavanol derivative" or Flavonoid* or Anthocyanin* or Catechin* or "Endothelial Nitric Oxide Synthase" or ENOS or Citrulline or Arginine or Antioxidant* or “beta vulgaris” or beetroot* or blackcurrant* or Ribes or blueberr* or cranberr* or “Vaccinium macrocarpon” or berry or grape* or vitis or pomegranate* or Punica* or cacao or cocoa or chocolate* or dark chocolate* or spinach* or “Spinacia oleracea” or red spinach* or watermelon* or Citrullus* or cherr* or “Prunus avium” or tea or "black tea*" or “green tea*”) **AND** TS=("Exercise Tolerance" or "Cardiorespiratory fitness" or "Physical endurance" or "Endurance Training" or “Cardiopulmonary exercise test” or "endurance performance" or Exhaustion or "Time-trial performance" or "time trial performance" or "cycling w/3 performance" or "running w/3 performance" or "swimming w/3 performance" or "rowing w/3 performance" or time w/3 fatigue or "aerobic power" or "aerobic capacity" or "aerobic performance" or "maximal oxygen uptake" or "intermittent exercise" or "repeated sprint" or "high-intensity exercise" or “high intensity exercise” or "Yo-Yo Intermittent Recovery Test" or "shuttle test")) **AND** LANGUAGE: (English)

**COMBINED (OR)**

TOPIC: (human? OR athlete?) **AND** TOPIC: ("Nitric oxide" OR nitrate? OR nitrite? OR polyphenol? OR flavanol? OR flavonoid? OR anthocyanin? OR catechin? OR endothelial AND nitric AND oxide AND synthase OR citrulline OR arginine OR antioxidant? OR almond? OR beetroot OR "Beetroot Juice" OR "Beta vulgaris" OR black?currant OR "Black tea extract" OR blueberry OR cacao OR cherry OR chocolate OR cranberry OR grape? OR "Grape seed extract" OR "Green tea" OR "Montmorency cherry" or "Montmorency tart cherry" OR nuts OR "Pomegranate" OR "Red Spinach Extract" OR "Tart cherry" OR "Vaccinium macrocarpon" OR watermelon) **AND** TOPIC: ("Exercise tolerance" OR "Cardiorespiratory fitness" OR "Physical endurance" OR "Endurance training" OR "Endurance performance" OR "Time?trial performance" OR "Cycling performance" OR "Running performance" OR "Swimming performance" OR "Rowing performance" OR exhaustion OR "Aerobic power" OR "Aerobic capacity" OR "Aerobic performance" OR "Oxygen uptake" OR "Intermittent exercise" OR "High-intensity exercise" OR "Repeated sprint" OR "Yo-Yo Intermittent Recovery Test" OR "Shuttle test")

**Scopus Search Strategy**

( TITLE-ABS-KEY ( human? OR athlete? OR runner? OR swimmer? OR rower? OR cyclist? ) **AND** TITLE-ABS-KEY ( "Nitric Oxide" OR "Nitric acid derivative" OR nitrate* OR nitrite* OR polyphenol* OR flavonol* OR "Flavanol derivative" OR flavonoid* OR anthocyanin* OR catechin* OR "Endothelial Nitric Oxide Synthase" OR enos OR citrulline OR arginine OR antioxidant* ) OR TITLE-ABS-KEY ( "beta vulgaris" OR beetroot* OR blackcurrant* OR ribes OR blueberr* OR cranberr* OR "Vaccinium macrocarpon" OR berry OR grape* OR vitis OR pomegranate* OR punica* OR cacao OR cocoa OR chocolate* OR "dark chocolate*" ) OR TITLE-ABS-KEY ( spinach* OR "Spinacia oleracea" OR "red spinach*" OR watermelon* OR citrullus* OR cherr* OR "Prunus avium" OR tea OR "black tea*" OR "green tea*" ) **AND** TITLE-ABS-KEY ( "Exercise Tolerance" OR "Cardiorespiratory fitness" OR "Physical endurance" OR "Endurance Training" OR "Cardiopulmonary exercise test" OR "endurance performance" OR exhaustion OR "Time-trial performance" OR "time trial performance" ) OR TITLE-ABS-KEY ( "cycling adj3 performance" OR "running adj3 performance" OR "swimming adj3 performance" OR "rowing adj3 performance" OR "time adj3 fatigue" OR "aerobic power" OR "aerobic capacity" OR "aerobic performance" ) OR TITLE-ABS-KEY ( "maximal oxygen uptake" OR "intermittent exercise" OR "repeated sprint" OR "high-intensity exercise" OR "high intensity exercise" OR "Yo-Yo Intermittent Recovery Test" OR "shuttle test" ) ) **AND** ( LIMIT-TO ( LANGUAGE , "English" ) )

**SportDiscus Search Strategy**

(human# OR athlete# OR runner# OR swimmer# OR rower# OR cyclist# ) **AND** ( "Nitric Oxide" OR "Nitric acid derivative" OR nitrate* OR nitrite* OR polyphenol* OR flavonol* OR "Flavanol derivative" OR flavonoid* OR anthocyanin* OR catechin* OR "Endothelial Nitric Oxide Synthase" OR enos OR citrulline OR arginine OR antioxidant* ) OR ( "beta vulgaris" OR beetroot* OR blackcurrant* OR ribes OR blueberr* OR cranberr* OR "Vaccinium macrocarpon" OR berry OR grape* OR vitis OR pomegranate* OR punica* OR cacao OR cocoa OR chocolate* OR "dark chocolate*" ) OR TITLE-ABS-KEY ( spinach* OR "Spinacia oleracea" OR "red spinach*" OR watermelon* OR citrullus* OR cherr* OR "Prunus avium" OR tea OR "black tea*" OR "green tea*" ) **AND** ( "Exercise Tolerance" OR "Cardiorespiratory fitness" OR "Physical endurance" OR "Endurance Training" OR "Cardiopulmonary exercise test" OR "endurance performance" OR exhaustion OR "Time-trial performance" OR "time trial performance" ) OR TITLE-ABS-KEY ( "cycling adj3 performance" OR "running adj3 performance" OR "swimming adj3 performance" OR "rowing adj3 performance" OR "time adj3 fatigue" OR "aerobic power" OR "aerobic capacity" OR "aerobic performance" ) OR TITLE-ABS-KEY ( "maximal oxygen uptake" OR "intermittent exercise" OR "repeated sprint" OR "high-intensity exercise" OR "high intensity exercise" OR "Yo-Yo Intermittent Recovery Test" OR "shuttle test" ))

*Search limited to English language and Academic Journal as Publication Type*
