# Supplementary material for: Effect of food sources of nitrate, polyphenols, L-arginine and L-citrulline on endurance exercise performance: a systematic review and meta-analysis of randomised controlled trials
Source: J Int Soc Sports Nutr. 2021 Dec 29;18:76. doi: 10.1186/s12970-021-00472-y (PMC8715640; doi:10.1186/s12970-021-00472-y)
Supplement: Supplementary file 2 — Additional file 2. Cochrane Risk of Bias Tool 2.0 Summary. Assessments of overall and domain-specific bias of included studies. [file 12970_2021_472_MOESM2_ESM.docx]

Noah MA d’Unienville ^a,b^_,_ Henry T Blake ^a,b^_,_ Alison M Coates ^a,b^_,_ Alison M Hill ^b,c^_,_ Maximillian J Nelson ^a,b^ & Jonathan D Buckley ^a,b^, ‘Effect of food sources of nitrate, polyphenols, L-arginine and L-citrulline on endurance exercise performance: a systematic review and meta-analysis of randomised controlled trials’_,_ *Journal of the International Society of Sports Nutrition*

^a^ Allied Health and Human Performance, University of South Australia, Adelaide, Australia

^b^ Alliance for Research in Exercise, Nutrition and Activity (ARENA), University of South Australia, Adelaide, Australia

^c^ Clinical and Health Sciences, University of South Australia, Adelaide, Australia

Corresponding Author: Noah M. A. d’Unienville - Contact email: Noah.D'Unienville@unisa.edu.au

**Online Resource 2:** Cochrane Risk of Bias Tool 2.0 Summary

| **Reference** | **Risk of Bias** | | | | | |
| --- | --- | --- | --- | --- | --- | --- |
|  | **RP** | **DII** | **MOD** | **MO** | **SRR** | **Overall Bias** |
| Abbey et al 2009 | ? | + | ? | + | ? | ? |
| Allen et al. 1998 | ? | - | + | + | ? | - |
| Allgrove et al. 2011 | ? | - | ? | + | ? | - |
| Areta et al. 2018 | ? | + | + | + | ? | ? |
| Aucouturier et al. 2015 | ? | - | + | + | ? | - |
| Bailey et al. 2009 | ? | - | + | + | ? | - |
| Bailey et al. 2015 | ? | + | + | + | ? | ? |
| Bailey et al. 2016 | ? | + | ? | + | ? | ? |
| Balsalobre-Fernandez et al. 2018 | ? | - | + | + | ? | - |
| Basta et al. 2013 | ? | + | + | + | ? | ? |
| Bell et al. 2014 | + | - | + | + | ? | - |
| Bell et al. 2015 | + | - | + | + | ? | - |
| Bentley et al. 2012 | ? | - | + | + | ? | - |
| Bernardi et al. 2018 | ? | - | ? | + | ? | - |
| Boorsma, Whitfield & Spriet 2014 | ? | - | + | + | ? | - |
| Bousetta et al. 2019 | ? | - | + | + | ? | ? |
| Braakhuis, Hopkins & Lowe 2014 | ? | + | + | + | ? | ? |
| Brandenburg et al. 2019 | ? | + | ? | + | ? | ? |
| Breese et al. 2013 | ? | - | ? | + | ? | - |
| Callahan et al. 2017 | ? | + | + | + | ? | ? |
| Cermak et al. 2012 | ? | + | + | + | ? | ? |
| Cermak, Gibala & van Loon 2012 | ? | - | + | + | ? | - |
| Chang et al. 2018 | + | + | + | + | ? | ? |
| Christensen et al. 2017 | ? | - | + | + | ? | - |
| Christensen, Nyberg & Bangsbo 2013 | ? | - | + | + | ? | - |
| Clifford et al. 2013 | ? | - | ? | + | ? | - |
| Cook et al. 2015 | ? | + | + | + | ? | ? |
| Crum, Barnes & Stannard 2018 | ? | + | ? | + | ? | ? |
| Cutrufello, Gadomski & Zavorsky 2015 | ? | - | + | + | ? | - |
| de Castro et al. 2019a | ? | - | + | + | ? | - |
| de Castro et al. 2019b | ? | - | + | + | ? | - |
| de Castro, de Assis Manoel, & Machado 2018 | ? | - | + | + | ? | - |
| Dean, Braakhuis & Paton 2009 | ? | + | + | + | ? | ? |
| Decroix et al. 2017 | ? | + | ? | + | ? | ? |
| Decroix et al. 2018 | + | - | + | + | ? | - |
| Deley et al. 2017 | ? | - | + | + | ? | - |
| Dowling et al. 1996 | + | + | + | + | ? | ? |
| Eichenberger et al. 2010 | ? | - | + | + | ? | - |
| Engels, Said & Wirth 1996 | ? | - | + | + | ? | - |
| Eschbach et al. 2000 | ? | - | ? | + | ? | - |
| Esen et al. 2019 | ? | - | + | + | ? | - |
| Esquius et al. 2019 | ? | + | ? | + | ? | ? |
| Flueck et al. 2019 | ? | + | ? | + | ? | ? |
| Gaamouri et al. 2019 | ? | + | + | + | ? | ? |
| Garnacho | ? | + | ? | + | ? | ? |
| Gelabert-Rebato et al. 2019 | ? | - | + | + | ? | - |
| Glaister et al. 2015 | ? | - | ? | + | ? | - |
| Gonzalez et al. 2019 | ? | + | ? | + | ? | ? |
| Handzik & Gleeson, 2013 | ? | + | ? | + | ? | ? |
| Hoon et al. 2014a | + | + | + | + | ? | ? |
| Hoon et al. 2014b | ? | + | + | + | ? | ? |
| Hsu et al. 2005 | ? | - | + | + | ? | - |
| Jonvik et al. 2018 | ? | - | + | + | ? | - |
| Jówko et al. 2018 | ? | + | + | + | ? | ? |
| Kalafati et al. 2010 | ? | + | ? | + | ? | ? |
| Keane et al. 2018 | ? | + | + | + | ? | ? |
| Kelly et al. 2013 | ? | + | ? | + | ? | ? |
| Kelly et al. 2014 | ? | + | ? | + | ? | ? |
| Kent et al. 2018 | ? | - | + | + | ? | - |
| Kern, Heslin & Rezende 2007 | ? | + | + | + | ? | ? |
| Knab et al. 2014 | ? | + | + | + | ? | ? |
| Kuo et al. 2014 | ? | + | + | + | ? | ? |
| Labonté et al. 2013 | ? | - | ? | + | ? | - |
| Lane et al. 2014 | + | + | ? | + | ? | ? |
| Lansley et al. 2011a | ? | + | + | + | ? | ? |
| Lansley et al. 2011b (JAP) | ? | - | ? | + | ? | - |
| Lowings et al. 2017 | ? | - | + | + | ? | - |
| Mach et al. 2010 | ? | - | ? | + | ? | - |
| Macleod et al. 2015 | ? | + | + | + | ? | ? |
| Martin et al. 2014 | ? | + | + | + | ? | ? |
| McQuillan et al. 2017a | ? | + | + | + | ? | ? |
| McQuillan et al. 2017b | ? | + | + | + | ? | ? |
| Montenegro et al. 2017 | ? | - | + | + | ? | - |
| Moore et al. 2017 | ? | + | + | + | ? | ? |
| Morgan, Barton & Bowtell 2019 | ? | + | + | + | ? | ? |
| Morris et al. 1996 | ? | - | ? | + | ? | - |
| Mosher et al. 2019 | ? | - | + | + | ? | - |
| Muggeridge et al. 2013 | ? | - | + | + | ? | - |
| Muggeridge et al. 2015 | + | - | ? | + | ? | - |
| Mumford et al. 2018 | ? | - | + | + | ? | - |
| Murphy et al. 2012 | ? | + | + | + | ? | ? |
| Murphy, Cook & Willams 2017 | ? | - | ? | + | ? | - |
| Nayebifar et al. 2016 | + | - | + | + | ? | - |
| Nieman et al. 2018 | ? | + | + | + | ? | ? |
| O'Connor et al. 2013 | + | + | + | + | ? | ? |
| Oh et al. 2010 | ? | + | + | + | ? | ? |
| Oskarsson & McGawley 2018 | ? | - | + | + | ? | - |
| Ostojic et al. 2008 | ? | + | + | + | ? | ? |
| Overdevest et al. 2018 | ? | - | + | + | ? | - |
| Patel, Brouner & Spendiff 2015 | ? | - | + | + | ? | - |
| Pawlak-Chaouch et al. 2019 | ? | - | + | + | ? | - |
| Peeling et al. 2015 | ? | - | + | + | ? | - |
| Perkins et al. 2015 | ? | - | + | + | ? | - |
| Pinna et al. 2014 | ? | - | ? | + | ? | - |
| Pospieszna et al. 2016 | ? | + | ? | + | ? | ? |
| Potter et al. 2019 | ? | - | + | + | ? | - |
| Rietschier et al. 2011 | ? | - | ? | + | ? | - |
| Rokkedal-Lausch et al. 2019 | ? | - | + | + | ? | - |
| Shanely et al. 2016 | ? | - | + | + | ? | - |
| Shannon et al. 2017 | ? | - | + | + | ? | - |
| Skarpanska-Stejnborn et al. 2006 | ? | - | + | + | ? | - |
| Tan et al. 2018 | ? | + | ? | + | ? | ? |
| Tarazona-Diaz et al. 2013 | ? | - | + | + | ? | - |
| Thompson et al. 2014 | ? | + | + | + | ? | ? |
| Thompson et al. 2016 | ? | + | ? | + | ? | ? |
| Thompson et al. 2017 | + | + | + | + | ? | ? |
| Thompson et al. 2018 | + | + | + | + | ? | ? |
| Torregrosa-García et al. 2019 | + | + | + | + | ? | ? |
| Toscano et al. 2015 | + | + | + | + | ? | ? |
| Trexler et al. 2014 | ? | + | ? | + | ? | ? |
| Ueberschlag et al. 2016 | + | + | + | + | ? | ? |
| Van Hoorebeke et al. 2016 | ? | - | + | + | ? | - |
| Vanhatalo et al. 2010 | ? | + | ? | + | ? | ? |
| Vasconcellos et al. 2017 | ? | + | + | + | ? | ? |
| Wasuntarawat et al. 2010 | ? | - | + | + | ? | - |
| Wilkerson et al. 2012 | ? | + | ? | + | ? | - |
| Willems et al. 2015 | ? | - | + | + | ? | - |
| Willems et al. 2016 | ? | - | + | + | ? | - |
| Wylie et al. 2013a | ? | + | ? | + | ? | ? |
| Wylie et al. 2013b | ? | - | + | + | ? | - |
| Wylie et al. 2016 | ? | + | ? | + | ? | ? |
| Wylie et al. 2019 | ? | - | + | + | ? | - |
| Yi et al. 2014 | ? | - | ? | + | ? | - |
