# Supplementary material for: Effect of food sources of nitrate, polyphenols, L-arginine and L-citrulline on endurance exercise performance: a systematic review and meta-analysis of randomised controlled trials
Source: J Int Soc Sports Nutr. 2021 Dec 29;18:76. doi: 10.1186/s12970-021-00472-y (PMC8715640; doi:10.1186/s12970-021-00472-y)
Supplement: Supplementary file 3 — Additional file 3. Study Characteristics and Summary Table. Description: Key characteristics and results of included studies. [file 12970_2021_472_MOESM3_ESM.docx]

Noah MA d’Unienville ^a,b^_,_ Henry T Blake ^a,b^_,_ Alison M Coates ^a,b^_,_ Alison M Hill ^b,c^_,_ Maximillian J Nelson ^a,b^ & Jonathan D Buckley ^a,b^, ‘Effect of food sources of nitrate, polyphenols, L-arginine and L-citrulline on endurance exercise performance: a systematic review and meta-analysis of randomised controlled trials’_,_ *Journal of the International Society of Sports Nutrition*

^a^ Allied Health and Human Performance, University of South Australia, Adelaide, Australia

^b^ Alliance for Research in Exercise, Nutrition and Activity (ARENA), University of South Australia, Adelaide, Australia

^c^ Clinical and Health Sciences, University of South Australia, Adelaide, Australia

Corresponding Author: Noah M. A. d’Unienville - Contact email: Noah.D'Unienville@unisa.edu.au

**Online Resource 3 -** Study Characteristics and Outcomes Table

| **Reference** | **Study design** | **Sample size (females); age; V̇O_2max_ ml.kg.min^-1^; description** | **Food Source, daily dose** | **NO Precursor Content (daily)** | **Days** | **Final dose-test interval** | **Test protocol (Mode)** | **Performance effects** | **NO synthesis effects^1^** | **Exercise Physiology** | **Antioxidant Effects** |
| --- | --- | --- | --- | --- | --- | --- | --- | --- | --- | --- | --- |
| **L-Citrulline** | | | | | | | | | | | |
| Bailey et al. 2016 | DB, CO | 8 (0); 22 ± 2; 52 ± 8; Recreationally active | Watermelon, 300 mL concentrated juice | ~3.4 g L-citrulline; 0.42 g L-arginine | 16 | 1.5 h | TTE - 70% Δ (cycling) | → TTE | ↑ NO_3_^-^ → SBP, DBP & MBP | ↑ Mod-INT tissue O_2_ index → BLa, V̇O_2_ uptake & kinetics |  |
| Cutrufello, Gadomski & Zavorsky 2015 | DB, CO | 22 (11); 20.8 ± 1.3; 55.3 ± 6.7; Intercollegiate athletes | Watermelon, 710 mL fresh juice | ~1 g L-citrulline | 1 | >1 h or >2 h | GXT - Modified Bruce protocol (incline walk/run) | → TTE (males) | → FMD | → V̇O_2max_ & AnT |  |
|  |  |  |  |  |  |  |  | → TTE (females) | → FMD | → V̇O_2max_ & AnT |  |
| Shanely et al. 2016 | ?, CO | 20; 48.5 ± 10.3; 51.5 ± 8.5; Competitive cyclists | Watermelon, 980 mL puree | 1.47 g L-citrulline; 0.47 g L-arginine | 14 | 0.2 h and during TT | 75 km TT (cycling) | → TTC | ↑ NO_3_^-^ post-TT & 1-h post-TT  → Pre-ex NO_3_^-^ | ↑ TT RPE → BGL & BLa |  |
| Tarazona-Diaz et al. 2013 | ?, CO | 7 (0); 22.7 ± 0.8; V̇O_2max_ N/A; Regular sport participants | Watermelon, 500ml unpasteurised juice | 1.17 g L-citrulline | 1 | 1 h | ITT - 8 x 30-secs, 1 min rest | → RPM | - |  |  |
| **Nitrate** | | | | | | | | | | | |
| Aucouturier et al. 2015 | SB, CO | 12 (0); 22.8 ± 3.1; 46.6 ± 3.4; Team sport athletes | Beetroot, 500 mL juice | 340 mg NO_3_^-^ | 3 | 3 h | ITTE - 15 secs at 170 % of PAP, 30 secs rest (cycling) | ↑ Total work | ↑ NO_3_^-^ & NO_2_^-^  → SBP, DBP, femoral artery diameter, blood velocity or flow rate | ↑Red blood cell concentration in active muscle |  |
| Bailey et al. 2009 | DB, CO | 8 (0); 26 ± 7; 49± 5; Recreationally active | Beetroot, 500 mL organic juice | 5.5 mmol NO_3_^-^ | 6 | NR  (day prior) | TTE - 70% Δ (cycling) | ↑ TTE | ↑ NO_2_^-^ ↓ SBP → DBP, MAP | ↑ Mod-INT muscle oxygenation  ↓ Rise in Mod-INT V̇O_2_; Sev-INT V̇O_2_ slow component and speed of V̇O_2_ uptake kinetics → Sev-INT muscle oxygenation; max or submax BLa, VE & RER |  |
| Bailey et al. 2015 | DB, CO | 7 (0); 21 ± 2; 51.38 (SD N/A); Recreationally active | Beetroot, 140 mL concentrated juice | 12.4 mmol NO_3_^-^ | 10 | 2.5 h | TTE - 80% Δ - 35 RPM (cycling) | → TTE | ↑ NO_2_^-^ ↓ DBP & SBP  → MAP | → Muscle HbO_2_ & V̇O_2_ kinetics |  |
|  |  |  |  |  |  |  | TTE @ 80% Δ - 115 RPM (cycling) | ↑ TTE |  | ↑ muscle HbO_2_; phase II V̇O_2_kinetics speed |  |
| Balsalobre-Fernandez et al. 2018 | DB, P | 12 (0); 25.8 ± 5.4; 70.7 ± 6.1; Elite runners (middle and long distance) | Beetroot, 70 mL concentrated juice | 6.5 mmol NO_3_^-^ | 15 | NR  (day prior) | GXT - 3 mins at 15, 17.1 and 20 km/h. Then 0.2 km/h faster every 12 secs (running) | → TTE | - | ↓ RPE → Leg stiffness, economy, V̇O_2max_, RER, HR & smO_2_ |  |
| Bernardi et al. 2018 | DB, CO | 10 (?); 24.9 ± 4.6; V̇O_2max_ N/A; Well-trained MMA athletes | Beetroot, 400 mL fresh juice | 9.3 mmoL NO_3_^-^ | 1 | 2 h | ITT: 20 x 6-sec sprints, 24 secs rest (cycling) | → MPO | - | - |  |
| Boorsma, Whitfield & Spriet, 2014 | DB, CO | 8 (0); 23.8 ± 5; 80 ± 5; Elite distance runners | Beetroot, 210 mL concentrated juice | 19.5 mmol NO_3_^-^ | 1 | 2.5 h | 1500 m TT (running) | → TTC | ↑ NOx | → Submax V̇O_2_, V̇CO_2_, RER, or HR |  |
|  |  |  | Beetroot, 140-210 mL concentrated juice | 13-19.5 mmol NO_3_^-^ | 8 |  |  | → TTC | ↑ NOx compared baseline and acute supplement |  |  |
| Breese et al. 2013 | DB, CO | 9 (5); 30 ± 6; 47.3 ± 7.8 ^a^; Recreationally active | Beetroot, 140 mL concentrated juice | 8 mmol NO_3_^-^ | 6 | 2.75 h | TTE - 70% Δ (cycling) | ↑ TTE | ↑ NO_2_^-^ | ↑ V̇O_2_ & HHb kinetics from Mod- to Sev-INT exercise.  → max, sub-max or kinetics of V̇O_2_, HHb, HR or BLa |  |
| Callahan et al. 2017 | DB, CO | 8 (0); 34 ± 7; 65.2 ± 4.2; Well-trained cyclists | Beetroot, 15 g beetroot crystals | 300 mg or ~5 mmol NO_3_^-^ | 3 | 1 h | 4 km cycling TT (cycling) | → TTC | ↑ NO_3_^-^ pre-TT, post-TT, 75 mins post-TT → NO_3_^-^ 60- or 165-mins pre-TT; NO_2_^-^ at any point | → pH, BLa, BGL & bicarbonate |  |
| Cermak et al. 2012 | DB, CO | 20 (0); 26 ± 1; 60 ± 1; Trained cyclists and triathletes | Beetroot, 140 mL concentrated juice | 8.7 mmol NO_3_^-^ | 1 | 2.5 h | ~60 min TT (cycling) | → TTC | ↑ NO_2_^-^ & NO_3_^-^ | → TT HR and post TT BLa |  |
| Cermak, Gibala & van Loon 2012 | DB, CO | 12 (0); 31 ± 3; 58 ± 2; Trained cyclists and triathletes | Beetroot, 140 mL concentrated juice | 8 mmol NO_3_^-^ | 6 | 3.5 h | 10 km TT (cycling) | ↓ TTC | ↑NO_3_^-^ → DBP, SBP | ↓ Submax V̇O_2_ → Submax V̇CO_2_, RER, energy expenditure, HR or RPE. Resting, submax or post-TT glucose, BLa or insulin. |  |
| Christensen et al. 2017 | DB, CO | 9 (0); 33 ± 11; 64±3; Trained cyclists | Beetroot, 150 mL juice | 9 mmol NO_3_^-^ | 1 | ~4.2 h | GXT-Cycling - Started at 40% PPO, then increased 25 W/min (cycling) | ↑ PPO | ↑ NO_3_^-^, NO_2_^-^ → MAP | → RER, BLa, max and submax V̇O_2_. No correlation to NO_2_^-^ & NO_3_^-^ changes. |  |
|  |  |  |  |  |  |  | GXT - Same protocol (hand-cycling) | → PPO |  | → RER, BLa, max and submax V̇O_2_. No correlation to NO_2_^-^ & NO_3_^-^ changes. |  |
|  |  | 8 (0); 27 ± 9; 46 ± 3; Recreationally active |  |  |  |  | GXT - (Cycling) | → PPO | ↑ NO_3_^-^ → MAP, NO_2_^-^ | → RER, BLa, max and submax V̇O_2_. No correlation to NO_2_^-^ & NO_3_^-^ changes. |  |
|  |  |  |  |  |  |  | GXT - Same protocol (hand-cycling) | → PPO |  | → RER, BLa, max and submax V̇O_2_. No correlation to NO_2_^-^ & NO_3_^-^ changes. |  |
| Christensen, Nyberg & Bangsbo 2013 | SB, CO | 8 (0); 29 ± 4; 72.1 ± 4.5; Elite cyclists | Beetroot, 500 mL juice | 500mg NO_3_^-^ | 6 |  | 400 kcal TT (cycling) | → MPO | ↑ NOx | → Submax V̇O_2_ steady-states or kinetics, RER & efficiency |  |
| de Castro et al. 2019a | DB, CO | 13 (0); 28.2 ± 3.0; 45.1 ± 5.8; Recreationally active runners | Beetroot, 420 mL fresh juice | 8.4 mmol NO_3_^-^ | 4 | 2 h | GXT - Initial speed of 8 km/h, increased 1 km/h every 3 min | ↑ TTE | - | ↑ V̇O_2max_ → Max BLa, HR, RPE or pre/post BGL |  |
|  |  |  |  |  |  |  |  | ↑ Peak velocity |  | → Max BLa, max HR, RPE or pre/post BGL |  |
| de Castro et al. 2019b | DB, CO | 14 (0); 27.8 ± 3.4; 45.4 ± 5.9; Recreationally active runners | Beetroot, 420 mL fresh juice | 8.4 mmol NO_3_^-^ | 4 | 2 h | 10 km TT (running) | → TTC | - | → Max BLa, max HR, RPE & pre/post glycogen |  |
| de Castro, de Assis Manoel, & Machado 2018 | ?, CO | 8 (8); 30.1 ± 5.7; V̇O_2max_ N/A; Untrained | Beetroot, 500 mL juice | 8.4 mmol NO_3_^-^ | 1 | 0.5 h | 3 km TT (running) | → TTC | - | ↑ TT max RPE  ↓Post-TT BGL  → Max HR, BLa & pre-TT BGL |  |
| Esen et al. 2019 | DB, CO | 10 (5); 22 ± 6; V̇O_2max_ N/A; Moderately trained swimmers | Beetroot, 140 mL concentrated juice | 8 mmol NO_3_^-^ | 3 | 3 h | 200 m front crawl TT, including diving start (swimming) | → TTC | ↑ NO_2_^-^ ↓ SBP  → DBP | →Post TT BLa |  |
| Flueck et al. 2019 | SB, CO | 14 (0); 28 ± 7; 33.9 ± 4.2^b^; Recreationally active, upper body trained | Beetroot, 140 mL concentrated juice | 6 mmol NO_3_^-^ | 1 | 3 h | 10 km TT (hand cycling) | → TTC | ↑ NO_2_^-^ & NO_3_^-^ | → Mean or max HR, O_2_ economy, or max RPE & BLa. |  |
|  |  | 8 (0); 40 ± 11; 38.6 ± 10.5^b^; National level paracyclists |  |  |  |  |  | → TTC | ↑ NO_2_^-^ & NO_3_^-^ | → Mean or max HR, Max RPE or BLa. |  |
| Garnacho-Castaño et al. 2018 | DB, CO | 12 (0); 39.3 ± 7.5; 54.8 ± 3.1; National or international level triathletes | Beetroot, 70 mL concentrated juice | 6.5 mmol NO_3_^-^ | 1 | 3.5 h | TTE - VT2 (cycling) | → TTE | - | ↑ VCO_2_ → HR, VO2, RER, VE |  |
| Glaister et al. 2015 | DB, CO | 14 (14); 31 ± 7; 52.3 ± 4.9; Well-trained cyclists and triathletes | Beetroot, 70 mL concentrated juice | 7.3 mmol NO_3_^-^ | 1 | 2.5 h | 20 km TT (cycling) | → TTC | ↑ NO_2_^-^ & NO_3_^-^ | → V̇O_2_, RPE, tissue oxygenation, iEMG, HR, RER or BLa |  |
| Handzik & Gleeson, 2013 | DB, CO | 14 (0); 22 ± 3; 63 ± 10; Well-trained endurance athletes | Beetroot, 140 mL concentrated juice | 8 mmol NO_3_^-^ | 1 | 1.75 h | TTE - 80% V̇O_2max_ (cycling) | → TTE | ↑ NO_2_^-^ & NO_3_^-^ (salivary) | ↓ RPE at 15 mins of TTE  → Mod or Sev-INT HR, V̇O_2_, V̇CO_2_, CHO/fat oxidation or RER. RPE at all other timepoints |  |
| Hoon et al. 2014a | DB, CO | 26 (0); 20.3 ± 1.4; V̇O_2max_ N/A; Trained cyclists | Beetroot, 140 mL concentrated juice | 4.1 mmol NO_3_^-^ | 1 | 2.5 h | 4 min TT (cycling) | → MPO | N/A (MBIs) |  |  |
|  |  |  |  |  |  | 1.25 h |  | → MPO |  |  |  |
| Hoon et al. 2014b | DB, CO | 10 (0); 20.6 ± 2.5; V̇O_2max_ N/A; Highly trained rowers | Beetroot, 70 mL concentrated juice | 4.2 mmol NO_3_^-^ | 1 | 2 h | 2000 m TT (rowing) | → TTC | N/A (MBIs) | N/A (MBIs) |  |
|  |  |  | Beetroot, 140 mL concentrated juice | 8.4 mmol NO_3_^-^ |  |  |  | → TTC | N/A (MBIs) | N/A (MBIs) |  |
| Jonvik et al. 2018 | DB, CO | 14 (0); 22 ± 4; V̇O_2max_ N/A; Elite (national) water polo players | Beetroot, 140 mL concentrated juice | 800 mg NO_3_^-^ | 5 | 3.5 h | ITT - 4 × 4 15-m sprints, with 5 secs active rest between sprints and 30 secs semiactive rest between sets | → TTC | ↑ NO_2_^-^ & NO_3_^-^ (salivary & plasma) | - |  |
| Kelly et al. 2013 | DB, CO | 9 (0); 22 ± 3; 54.5 ± 7.5; Recreationally active | Beetroot, 500 mL juice | 8.2 mmol NO_3_^-^ | 7-12 | 2.5 h | TTE - 60% Δ (cycling) | ↑ TTE | ↑ NO_2_^-^ ↓SBP  → DBP & MAP | → V̇O_2_ phase II TC, primary amplitude or slow component; V̇O_2,_ HR & BLa at exhaustion. |  |
|  |  |  |  |  |  |  | TTE - 70% Δ (cycling) | ↑ TTE |  | → V̇O_2_ phase II TC, primary amplitude or slow component; V̇O_2,_ HR & BLa at exhaustion. |  |
|  |  |  |  |  |  |  | TTE - 80% Δ (cycling) | ↑ TTE |  | → V̇O_2_ phase II TC, primary amplitude or slow component; V̇O_2,_ HR & BLa at exhaustion. |  |
|  |  |  |  |  |  |  | TTE - 100% Δ (cycling) | ↑ TTE |  | → V̇O_2_phase II TC, primary amplitude or slow component; V̇O_2,_ HR & BLa at exhaustion. |  |
| Kelly et al. 2014 | DB, CO | 12 (0); 22 ± 4; 58.3 ± 6.3; Active | Beetroot, 140 mL concentrated juice | 8.4 mmol NO_3_^-^ | 3 | 2.5 h | TTE @ 75% Δ (cycling) | → TTE | ↑ NO_3_^-^ (at rest), NO_2_^-^ (at rest, during exercising and recovery) ↓ SBP  → DBP & MAP | → Mod or Sev-INT HR, arterial O_2_ saturation, HHb, HbO_2_, total Hb, end- V̇O_2_, V̇O_2_ phase II responses, primary amplitude or slow component |  |
| Kent et al. 2018 | DB, CO | 12 (0); 26.6 ± 4.4; 65.8 ± 5.5; Trained cyclists and triathletes | Beetroot, 70 or 140 mL concentrated juice | 6.5 or 13 mmol NO_3_^-^ | 3 | 2.5 h | 14 kJ/kg (50-60 min) TT (cycling) | → TTC | ↑ NO_2_^-^ & NO_3_^-^ (salivary) | → HR or RPE |  |
| Lane et al. 2014 | DB, CO | 24 (12); 29.5 ± 6.5; 66.0 ± 4.9; Competitive cyclists and triathletes | Beetroot, 140 mL concentrated juice | 8.4 mmol NO_3_^-^ | 1 | 2.2 h | 43.83 km (males) or 29.35 km (female) simulated TT (cycling) | → TTC (males) | ↑ NO_2_^-^ & NO_3_^-^.   NO_3_^-^ but not NO_2_^-^ ↑ after added dose | → TT HR or RPE |  |
|  |  |  |  |  |  |  |  | → TTC (females) | ↑ NO_2_^-^ & NO_3_^-^.   NO_3_^-^ but not NO_2_^-^ ↑ after added dose | → TT HR or RPE |  |
| Lansley et al. 2011a | DB, CO | 9 (0); 21 ± 4; 56.0 ± 5.7; Trained cyclists | Beetroot, 500 mL organic juice | 6.2 mmol NO_3_^-^ | 1 | 2.75 h | 4 km TT (cycling) | ↓ TTC | ↑ NO_2_^-^ ↓ SBP  → DBP & MAP | → TT mean & peak V̇O_2_ |  |
|  |  |  |  |  |  |  | 16.1 km TT (cycling) | ↓ TTC |  | → TT mean & peak V̇O_2_ |  |
| Lansley et al. 2011b (JAP) | DB, CO | 9 (0); 22 ± 4; 55 ± 7; Active | Beetroot, 500 mL organic juice | 6.2 mmol NO_3_^-^ | 4 | 3.5 h | TTE at 75% Δ (running) | ↑ TTE | ↑ NO_2_^-^ ↓ SBP  → DBP & MAP | ↓Mod-INT V̇O_2_slow component amplitude, walking V̇O_2_ & V̇O_2_ at exhaustion  → Phase II TC, primary or slow phase amplitude, HR or BLa. Mod-INT HR or BLa. |  |
| Lowings et al. 2017 | DB, CO | 10 (5); 20 ± 1; V̇O_2max_ N/A; Trained, competitive swimmers | Beetroot, 140 mL concentrated juice | 12.5 mmol NO_3_^-^ | 1 | >3.1 h | 168 m backstroke TT (swimming) | → TTC | ↑ FENO | → Pre/post-TT BLa |  |
| Macleod et al. 2015 | DB, CO | 11 (0); 29.3 ± 5.1; 67.5 ± 5.8; Trained cyclists | Beetroot, 70 mL concentrated juice | 6.5 mmol NO_3_^-^ | 1 | 2.5 h | 10 km TT (cycling) | → TTC | → NO_2_^-^ & FENO → DBP & SBP | → TT V̇O_2_ or pulse O_2_ saturation. Submax economy, VE, HR, pulse O_2_ saturation |  |
| McQuillan et al. 2017a | DB, CO | 9 (0); 27 ± 9; 68 ± 3; Highly trained cyclists | Beetroot, 140 mL concentrated juice | 8 mmol NO_3_^-^ | 3 | 2.5 h | 4 km TT (cycling) | → TTC | → NO_2_^-^ (serum) | → Submax O_2_ economy |  |
|  |  |  |  |  | 6 |  |  | → TTC | → NO_2_^-^ (serum) | → Submax O_2_ economy |  |
| McQuillan et al. 2017b | DB, CO | 8 (0); 26 ± 8; 63 ± 4 mL; Well-trained cyclists | Beetroot, 70 mL concentrated juice | 4 mmol NO_3_^-^ | 6 | ~2.25 h | GXT - 3 mins at 50 W, then increased 20 W/min | → TTE | N/A (MBIs) | N/A (MBIs) |  |
|  |  |  |  |  | 8 | ~2.5 h | 4km TT (cycling) | → TTC |  |  |  |
| Mosher et al. 2019 | DB, CO | 11 (?); 36 ± 9; 60.8 ± 7.4; Trained cyclists | Beetroot, 140 mL concentrated juice | 12.8 mmol NO_3_^-^ | 2 | ~3.1 h | 40 km TT (cycling) | → TTC | - | → Mean HR, V̇O_2_, BLa, RPE, pH & HCO_3_ |  |
| Muggeridge et al. 2013 | SB, CO | 8 (0); 31 ± 15; 49.0 ± 6.1; Trained kayakers | Beetroot, 70 mL concentrated juice | 5 mmol NO_3_^-^ | 1 | 3.5 h | 1 km TT (kayaking) | → TTC | á NO_2_^-^ & NO_3_^-^;  Δ NO2- inversely related to baseline NO2- → SBP & DBP | ↓ Submax and TT mean V̇O_2_ → TT V̇O_2peak_. |  |
| Murphy et al. 2012 | DB, CO | 11 (6); 25 ± 4; V̇O_2max_ N/A; Recreationally fit | Beetroot, 200g baked beetroot | >500mg NO_3_^-^ | 1 | 1 h | 5km TT (running) | → Mean velocity | → SBP & DBP | ↓RPE in first mile → HR or RPE at other time points |  |
| Oskarsson & McGawley 2018 | DB, CO | 9 (2); 30.6 ±; 58.2 ± 7.7; Recreationally to well-trained runners | Beetroot, 70 mL concentrated juice | 7.3 mmol NO_3_^-^ | 1 | ~2.9 h | 1 km TT (running) | → TTC | - | → Submax V̇O_2_, efficiency, RER, HR, or RPE; max HR, BLa & RPE |  |
| Pawlak-Chaouch et al. 2019 | SB, CO | 9 (0); 21.7 ± 3.7; 71.1 ± 5.2; Elite track and field athletes and triathletes | Beetroot, 500 mL juice | 340 mg (5.2 mmol) NO_3_^-^ | 3 | 3 h | ITTE - 15 secs at 170% W_max_, 30 secs passive rest (cycling) | → Total work | ↑ NOx (pre & post-test) Δ NOx not related to Δ performance or Δ V̇O_2_ | → V̇O_2_, V̇CO_2_, VE, HR, HbO_2_, HHb or THb |  |
| Peeling et al. 2015 | SB, CO | 6 (0); 24.7 ± 3.0; 57.15 ± 2.8; National-level kayakers | Beetroot, 70 mL concentrated juice | 4.8 mmol NO_3_^-^ | 1 | 3 h | 4 min TT (kayaking) | → Distance | - | ↓ TT V̇O_2_ → TT BLa, RPE & HR. |  |
| Pinna et al. 2014 | ?, CO | 14 (0); 34.7 ± 7.5; 42.7 ± 2.6; Moderately trained swimmers | Beetroot, 500 mL organic juice | 5.5 mmol NO_3_^-^ | 6 | NR | GXT - Dynamometer-controlled resistance via elastic rope (swimming) | → Max resistance | - | ↑ AnT  ↓ "aerobic energy cost" at AnT → V̇O_2_, V̇CO_2_, VE or HR at AnT or max |  |
| Rokkedal-Lausch et al. 2019 | DB, CO | 12 (0); 29.1 ± 7.7; 66.4 ± 5.3; Well-trained cyclists | Beetroot, 140 mL concentrated juice | 12.4 mmol NO_3_^-^ | 7 | 2.75 hours | 10 km TT (cycling) | → TTC | á: NO_2_^-^ & NO_3_^-^. Changes not correlated with performance | ↑ TT mean V̇O_2_, VE, & V̇CO_2_ → Max HR, RER & V̇O_2;_ TT mean RER; changes in HbO_2_, HHb & Thb |  |
| Shannon et al. 2017 | DB, CO | 8 (0); 28.3 ± 5.8; 62.3 ± 8.1; Trained runners and triathletes | Beetroot, 140 mL concentrated juice | 12.5 mmol NO_3_^-^ | 1 | 3 h | 1.5 km TT (running) | ↓ TTC | ↑ NO_2_^-^ pre/post TT → MAP | ↑ Post-TT BLa →TT V̇O_2_, V̇CO_2_or RER; Post-TT HR and sessional RPE |  |
|  |  |  |  |  |  |  | 10 km TT (running) | → TTC |  | →TT V̇O_2_, V̇CO_2_ or RER; Post-TT BLa, HR and sRPE |  |
| Tan et al. 2018 | DB, CO | 12 (0); 21 ± 1; 45 ± 4; Recreationally active | Beetroot, 140 mL concentrated juice | 12.4-18.6 mmol NO_3_^-^ | 3 | 1 h | 100 kJ TT (cycling) | → TTC | ↑ NO_3_^-^ & NO_2_^-^ at rest and during submaximal exercise | ↓ Rise in V̇O_2_ and decrease in muscle glycogen across preload → Preload RER, HR, VE, BGL & BLa; TT max HR & BLa, or mean V̇O_2_ & BGL. |  |
|  |  |  |  | 12.4 mmol NO_3_^-^ |  | 3.5 h |  | → TTC | ↑ NO_3_^-^ & NO_2_^-^ at rest and during submaximal exercise | → Preload V̇O_2_, RER, HR, VE, BGL, BLa, & muscle glycogen; TT max HR & BLa, or mean V̇O_2_ & BGL. |  |
| Thompson et al. 2014 | DB, CO | 16 (0); 24 ± 4; 47.3 ± 6.3; Recreationally active | Beetroot, 450 mL organic juice | 5 mmol NO_3_^-^ | 1 | 2.7 h | TTE - 90% V̇O_2peak_ (cycling) | ↑ TTE | ↑ Pre & post-exercise NO_2_^-^, post-exercise SBP  → Pre-exercise DBP, MAP at all times | ↑ RER across GXT, maintenance of muscle oxygenation, post-ex BLa ↓ Drop in muscle HHb  → Peak or submax V̇O_2_ |  |
| Thompson et al. 2016 | DB, CO | 36 (0); 24 ± 4; V̇O_2max_ N/A; Local and university level competition team sport athletes | Beetroot, 70 mL concentrated juice | 6.4 mmol NO_3_^-^ | 4 | ~2.6 h | IGXT - Yo-Yo IR1 running) | ↑ Total distance | ↑ NO_3_^-^ & NO_2_^-^ ↓SBP  → MAP & DBP | - |  |
| Thompson et al. 2017 | DB, P | 24 (12); 24.5 ± 7; 43.8 ± 13.4; Recreationally active in team and/or endurance sports | Beetroot, 140 mL concentrated juice | 12.8 mmol NO_3_^-^ | 28 | 2.75 h | GXT (cycling) | ↑ PPO | ↑ NO_3_^-^ & NO_2_^-^ ↓SBP  → MAP & DBP | ↓ Mod-INT V̇O_2_ → V̇O_2peak_ or V̇O_2_at GET |  |
|  |  |  |  |  |  | 2.9 h | TTE @ 85% Δ (cycling) | → TTE |  | ↑ Muscle glycogen at exhaustion  ↓ rise BLa during 3 mins of sev-INT |  |
| Thompson et al. 2018 | DB, P | 20 (8); 23.9 ± 5.0; 44.1 ± 6.3; Recreationally active in team and/or endurance sports | Beetroot, 140 mL concentrated juice | 12.8 mmol NO_3_^-^ | 28 | 2.75 h | GXT - 3 mins at 20 W, then increased 30 W/min (cycling) | → PPO | ↑ NO_2_^-^ ↓(Δ) SBP and DBP | ↑ V̇O_2peak_ (Δ) ↓ Mod-INT V̇O_2_ |  |
|  |  |  |  |  |  | 2.9 h | TTE - 85% Δ (cycling) | ↑ TTE | ↑Plasma NO_2_^-^ ↓ SBP & DBP (Δ) | ↓ BLa after 1 min Sev-INT → BLa at exhaustion; muscle PCr, ATP, glycogen or phenotype |  |
| Vanhatalo et al. 2010 | DB, CO | 8 (3); 29 ± 6; 46.8 ± 11.7; Active | Beetroot, 500 mL organic juice | 5.2 mmol NO_3_^-^ | 1 | 3.1 h | GXT - Workload increased by 1 W every 2 secs (cycling) | → PPO | ↑ NO_2_^-^ ↓SBP & MAP → DBP | ↓ Mod-INT V̇O_2_ amplitude.  → V̇O_2max_, GET V̇O_2_ or power; max V̇CO_2_, RER, VE, HR, BLA & BGL. |  |
|  |  |  |  |  | 5 |  |  | → PPO | ↑ NO_2_^-^ →SBP, DBP & MAP | ↓ Mod-INT V̇O_2_ & V̇O_2_amplitude.  → V̇O_2max_, GET V̇O_2_; max V̇CO_2_, RER, VE, HR, BLA & BGL. |  |
|  |  |  |  |  | 15 |  |  | ↑ PPO | ↑ NO_2_^-^ ↓SBP & MAP → DBP | ↑ GET power ↓ Mod-INT V̇O_2_ & V̇O_2_ amplitude.  → V̇O_2max_, GET V̇O_2_; max V̇CO_2_, RER, VE, HR, BLA & BGL. |  |
| Vasconcellos et al. 2017 | DB, CO | 25 (11); 35.8 ± 6.2; 59.6 ± 4.7; Active runners | Beetroot, 100 g beetroot-based gel | 9.9 mmol NO_3_^-^ | 1 | ~1.5 h | TTE - 70% Δ (cycling) | → TTE | ↑Urine NO_3_^-^ & NO_2_^-^ at rest and immediately post-exercise → SBP or DBP at any time | ↓ BGL post-TTE and 20 mins post-TTE → Mod-INT or peak V̇O_2,_ RQ, HR & RPE; Post-ex BLa |  |
| Wilkerson et al. 2012 | DB, CO | 8 (0); 31 ± 11; 63 ± 8; Well-trained cyclists | Beetroot, 500 mL organic juice | 6.2 mmol NO_3_^-^ | 1 | 2.5 h | 50 km TT (cycling) | → TTC | ↑NO2 → SBP, DBP, & MAP. NO2 & TT changes correlated (r = -0.83) | ↑ Efficiency (MPO/ V̇O_2_) → TT V̇O_2_, BLa, HR or RPE. |  |
| Wylie et al. 2013a | DB, CO | 10 (0); 22 ± 5; V̇O_2max_ N/A; Recreationally active | Beetroot, 70 mL concentrated juice | 4.2 mmol NO_3_^-^ | 1 | 2.8 h | TTE - 75% Δ (cycling) | → TTE | ↑NO_2_^-^ & NO_3_^-^ | → Mod or Sev-INT V̇O_2_, V̇O_2_ amplitude or increase, HR, BLa, VE, V̇CO_2_& RER |  |
|  |  |  | Beetroot, 140 mL concentrated juice | 8.4 mmol NO_3_^-^ |  |  |  | ↑ TTE | ↑NO_2_^-^ & NO_3_^-^ | → Mod or Sev-INT V̇O_2_, V̇O_2_amplitude or increase, HR, BLa, VE, V̇CO_2_& RER |  |
|  |  |  | Beetroot, 280 mL concentrated juice | 16.8 mmol NO_3_^-^ |  |  |  | ↑ TTE | ↑NO_2_^-^ & NO_3_^-^ | ↓ Mod-INT V̇O_2_ → Sev-INT V̇O_2_; Mod or Sev-INT V̇O_2_ amplitude or increase, HR, BLa, VE, V̇CO_2_& RER |  |
| Wylie et al. 2013b | DB, CO | 14 (0); 22 ± 2; 52 ± 7; Recreational team-sport players | Beetroot, 210-280 mL concentrated juice | 12.3 - 16.4 mmol NO_3_^-^ | 2 | 1.6 h | IGXT - Yo–Yo IR1 (running) | ↑ Total distance | ↑NO_2_^-^ & NO_3_^-^ | ↓ BGL across warm-up & Yo-Yo IR1 → Submax or max HR & BLa |  |
| Wylie et al. 2016 | DB, CO | 10 (0); 21 ± 1; 58 ± 8; Recreational team-sport players | Beetroot, 140 mL concentrated juice | 8.2 mmol NO_3_^-^ | 3 | 2.7 h | ITT - 24 x 6-sec sprints, with 24 secs passive recovery (cycling) | → MPO | ↑NO_2_^-^ | ↑ RER during work and rest periods; Greater rise in BLa from sprint 22  → V̇O_2_ or V̇CO_2_ |  |
|  |  |  |  |  | 5 |  | ITT - 6 x 60-sec efforts, with 40 secs recovery at 20 W, 20 secs passive recovery (cycling) | → MPO | ↑NO_2_^-^ | → BLa changes; V̇O_2_, V̇CO_2_or RER during work or rest periods. |  |
| Wylie et al. 2019 | SB, CO | 12 (5); 26.7 ± 10.0; 46.0 ± 6.8; Recreationally active | Beetroot, 140 mL concentrated juice | 12.8 mmol NO_3_^-^ | 1 | 2.1 h | TTE @ 70% Δ (cycling) | → TTE | ↑Plasma and muscle NO_2_^-^ & NO_3_^-^_._ | → V̇O_2_ at exhaustion during Sev-INT |  |
| Gonzalez et al. 2019 | DB, CO | 17 (8); 22.5 ± 3.7; V̇O_2max_ N/A; Recreationally active | Red spinach, 1 g powdered extract | 90 mg NO_3_^-^ | 8 | 1 h | 4km TT (cycling) | ↓ TTC | → Resting SBP & DBP, post-TT SBP ↓ post-TT DBP | → RPE, muscle pain & post-TT HR |  |
| Moore et al. 2017 | DB, CO | 14 (7); 23.7 ± 3.2; 40.9 ± 7.2; Recreationally active | Red spinach, 1 g powdered extract | 90 mg NO_3_^-^ | 1 | ~1.1-1.3 h | GXT - Bruce protocol (incline walk/run) | → TTE | ↑NO_3_^-^  → NO_2_^-^ | ↑ V̇O_2_at VT → V̇O_2peak_, time to VT |  |
| Muggeridge et al. 2015 | DB, CO | 9 (0); 36 ± 6; 53.1 ± 4.4; Trained cyclists and triathletes | Swiss chard and rhubarb, 120 mL extract-based gel | 8.1 mmol NO_3_^-^ | 1 | ~3.4 h | 16.1 km TT (cycling) | → TTC | ↑NO_3_^-^  → DBP, SBP & MAP | → Submax HR & V̇O_2_ |  |
| **Polyphenol** | | | | | | | | | | | |
| Yi et al. 2014 | ?, CO | 10 (0); 22.3 ± 1.6; 70.3 ± 4.6; Professional, trained cyclists and triathletes | Almonds, 75 g raw whole almonds (American) | ~2 g arginine | 28 | NR | 20-min TT (cycling) | → Total distance | →NOx | → Submax RPE; TT mean HR, RPE, V̇O_2_, energy expenditure & CHO/fat utilisation | ↑ TAC → GPx, SOD, MDA, BUN & CK |
| Basta et al. 2013 | DB, P | 18 (0); 20.9 ± 1.12; V̇O_2max_ N/A; National-level rowers | *Aloe Arborescens*, 0.25 mL extract |  | 28 | NR | 2,000 m TT (rowing) | → TTC | - | → Min or max BLa | ↑ TAC ↓ TBARS → GPx, SOD & CK |
| Hsu et al. 2005 | DB, CO | 13 (0); 23±1.6; 45.1±5.0; Active | American ginseng, 1600 mg |  | 28 | NR | TTE at 80% V̇O_2max_ (running) | → TTE | - | ↓ BLa at 15 & 30 mins of, & 120 mins after TTE → BLa immediately post-TTE | ↓ CK |
| Morris et al. 1996 | DB, CO | 8 (1); 27.2±4.8; 48±8; Mostly recreationally active | American ginseng, 8 or 16mg/kg (618 or 1235mg) extract |  | 7 | NR | TTE - 75% V̇O_2max_ (cycling) | → TTE |  | → RPE or rectal temperature, plasma FFA during or after exercise |  |
| Nieman et al. 2018 | ?, CO | 20 (6); 39.0 ± 8.4; 46.9 ± 6.0; Trained cyclists | Banana, Cavendish bananas - 0.4g/kg CHO |  | 1 | Consumed during TT | 75 km TT (cycling) | → TTC | - | → Mean V̇O_2_, HR, RPE or RER @ 55km |  |
|  |  |  | Banana, Mini-yellow bananas - 0.4g/kg CHO |  |  |  |  | → TTC | - | → Mean V̇O_2_, HR, RPE or RER @ 55km |  |
| Lansley et al. 2011b | DB, CO | 9(0); 22 ± 4; 55 ± 7; Active | Beetroot (NO_3_^-^-depleted), 500 mL organic juice |  | 4 |  | TTE - 75% Δ (running) | → TTE | → NO_2_-, SBP, DBP & MAP | → Walking, Mod- or Sev-INT V̇O_2_, V̇O_2_ Phase II TC, primary or slow phase amplitude, HR or BLa, HR or BLa. |  |
| Montenegro et al. 2017 | DB, CO | 22 (13); 38±11.3; 50.4±7.7; Recreationally competitive triathletes | Beetroot (NO_3_^-^-depleted), 100mg betalain rich concentrate |  | 7 | ~2.8 h | 10 km TT (running) | ↓ TTC | → Resting & exercise BP | → Submax HR, V̇O_2_%, & RER, or post-TT BLa. | ↓ CK |
| Mumford et al. 2018 | DB, CO | 24 (0); 28.8 ±9.8; V̇O_2max_ N/A; Trained cyclists | Beetroot (NO_3_^-^-depleted), 100 mg betalain-rich concentrate |  | 7 | ~2.3 h | 30-min TT (cycling) | → Total Distance | →FMD & NOx | ↑ Efficiency at 25-30 mins of TT → BLa, erythropoietin, pH, HR, V̇O_2_, SV, TPR & Q | → TBARS |
| Van Hoorebeke et al. 2016 | DB, CO | 15 (0); 25.3 ± 5.4; 55.6 ± 3.7; Recreationally-competitive runners | Beetroot (NO_3_^-^-depleted), 100 mg betalain-rich concentrate |  | 7 | 3 h | 5 km TT (running) | ↓ TTC | - | ↓Preload HR, BLa and RPE; TT RPE → Preload V̇O_2_, RER, %CHO/fat, BGL; TT mean HR, BLa, BGL | → LDH & CK |
| Skarpanska-Stejnborn et al. 2006 | SB, P | 19 (0); 20.5±1.3; 66.6±7.3; National rowers | Blackcurrant, 750 mg ground | 25.5mg GAE | 42 | NR | 2km TT (rowing) | → TTC | - | → Max BLa & V̇O_2max_, mean HR or VE | ↑ TAC ↓ GPx, SOD & TBARS |
| Brandenburg et al. 2019 | DB, CO | 14 (?); 31.3 ± 10.3; 51.1 ± 6.2; Healthy runners | Blueberry, 72 g freeze-dried powder | TPC - 2,433 mg, 1,008 mg anthocyanins | 2 | ~3 h | 8 km TT (running) | → TTC | - | → Mean or max HR & RPE; BLa at 5, 15 & 30 mins post-TT | → Uric acid |
|  |  |  |  |  | 4 |  |  | → TTC | - | ↓ BLa 5 mins post-TT → Mean or max HR & RPE | → Uric acid |
| Gaamouri et al. 2019 | DB, P | 23 (11); 21.9 ± 1.2; V̇O_2max_ N/A; National level taekwondo athletes | Carob, 40 g powder | TPC - 208 mg: 14.4 mg flavonoids | 42 |  | IGXT - Yo-Yo IRL1 (running) | ↑ Maximal velocity | - | ↑ Max RPE → Max HR |  |
| Pospieszna et al. 2016 | DB, CO | 11 (11); 20.9 ± 1.3; V̇O_2max_ N/A; University league level swimmers | Chokeberry and beetroot, 500 mL mixed juice | TPC - 1616 mg: 149 mg anthocyanins | 8 | >2.2 h | 800 m TT (swimming) | → TTC | → SBP & DBP | → Max HR |  |
| Overdevest et al. 2018 | DB, P | 39 (0); 23 ± 1.9; 57.1 (SD N/A) ^a^; Trained (unspecified mode) | Citrus, 500 mg citrus flavonoid | ≥ 450 mg hesperetin | 28 | NR | 10 min TT (cycling) | → MPO | - | ↑ TT Efficiency → TT mean HR & V̇O_2_ |  |
| Allgrove et al. 2011 | SB, CO | 20 (0); 22 ± 4; 53.1 ± 7.0; Active | Cocoa, 80 g dark chocolate | 31.2 mg catechin; 77.4 mg epicatechin | 15 | 3.5 h | TTE - 90% V̇O_2max_ (cycling) | → TTE | - | → Submax HR, RPE, V̇O_2_ & RER | ↓ F2-Isoprostanes |
| Decroix et al. 2017 | DB, CO | 12 (0); 30 ± 3; 63.0 ± 3.5; Well-trained cyclists | Cocoa, 12 g cocoa powder | 900 mg flavanols | 7 | 1.7 h | 20-min TT (cycling) | → TTC |  | → TT RPE, BGL, BLa & HR | ↑ TAC → MDA |
| Decroix et al. 2018 | DB, CO | 14 (0); 30.7 ± 3.1; 62.9 ± 5.8; Well-trained cyclists | Cocoa, 1765 mg cocoa extract | 530 mg total flavanols | 7 | 2 h | ~30-min TT (cycling) | → Total work | ↑ FMD - Unrelated to change in NO_2_^-^ or NO_3_^-^ → NO_2_^-^, NO_3_^-^ & MAP | → Submax or TT muscle oxygenation, SaO_2_, BLa, RPE & HR | ↓ MDA → TAC & Uric acid |
| Patel, Brouner & Spendiff 2015 | ?, CO | 9 (0); 21 ± 1; 41.89 ± 5.4; Moderately trained (unspecified mode) | Cocoa, 40 g dark chocolate |  | 14 | NR | 2-min TT (cycling) | ↑ Total distance | → SBP & DBP | ↑ GET power → V̇O_2max_; submaximal BLa, HR, RER or V̇O_2_ |  |
| Ostojic et al. 2008 | DB, P | 20 (6); 22.1 ± 2.7; 41.4 (SD N/A) ^a^ ; College athletes and physical education students | Coffeeberry, 800 mg coffeeberry formulation capsules |  | 28 | NR  (day prior) | GXT - Multistage 20 m shuttle test (running) | → Predicted V̇O_2max_ | - |  | ↑ TAC |
| Labonté et al. 2013 | DB, CO | 12 (2); 22±2.4; V̇O_2max_ N/A; 9 Elite speed skaters, 2 cyclists and 1 cross-country skier | Cranberries and grape seeds, 800mg CranLoad™ | TPC - 800 mg | 1 | 1 h | 3 km TT (cycling) | → TTC | ↑ FMD (not in same athletes) | ↓ BLa prior, post and 2.5min post-TT; HR 2 & 5 mins post-TT → Max HR; BLa 5 mins post-TT |  |
| Chang et al. 2018 | SB, P | 28 (0); 22.9 ± 2.3; 56.8 ± 11.1; Recreationally active runners | Danggui Buxue Tang, 7.5 g | 34.13 mg flavonoids, 0.9 mg phenolic acids | 11 | NR | 13 km TT (running) | ↓ TTC | - | - | → Catalase, SOD, MDA & LDH |
| Oh et al. 2010 | DB, CO | 20 (0); 20.9 ± 2.0; 49.7 ± 7.4; Active | *Ecklonia Cava*, 72 mg of *Ecklonia Cava* polyphenols (ECP) | 72 mg of ECP | 1 | 1.1 h | GXT - Modified Bruce protocol (incline walk/run) | ↑ TTE |  | ↑ BGL Post-TTE & 3 mins post → Submax or max V̇O_2_, HR or RER; BGL at 5, 15, 30 or 60 mins post-TTE; Bla post-TTE |  |
| Bentley et al. 2012 | DB, CO | 9 (0); 35 ± 10; 63 ± 11; Well-trained cyclists | French maritime pine, 360 mg Pycnogenol (in 150 mL Lactaway) |  | 1 | 4 h | TTE - 95% PPO (cycling) | → TTE | - | NA (MBIs) |  |
| Clifford et al. 2013 | DB, CO | 9 (0); 32.1 ± 11.2; 52.4 (SD N/A); Moderately trained cyclists and triathletes | French maritime pine, 120 mg Pycnogenol and 600 mg citrus 'bioflavanoids' | 600 mg flavanoids | 3 |  | 20 km TT (cycling) | → TTC | - | → Submax V̇O_2_, BLa, efficiency, HR or RER; TT HR & BLa |  |
| Mach et al. 2010 | DB, CO | 13 (0); 29.5 ± 6.6; 57.3 ± 5.4; Mix of trained cyclists & untrained | French maritime pine, 360 mg Pycnogenol (in 150 mL Lactaway) |  | 1 | 3.2 h | TTE - 95% PPO (cycling) | ↑ TTE | - | ↑ V̇O_2peak_ in trained subjects  → BLa or HR | → TBARS |
| Nayebifar et al. 2016 | ?, P | 16 (16); 22.1 ± 3.3; V̇O_2max_ N/A; Inactive | Ginger, 3 g ginger tablets |  | 70 | NR | GXT - Bruce protocol (incline walk/run) | → Predicted V̇O_2max_ | - | - |  |
| O'Connor et al. 2013 | DB, P | 40 (19); 19.9 ± 2.0; 42.0 ± 8.7; Recreationally active | Grape, 46 g of freeze-dried grape powder (mixed varieties) | 0.1 mg resveratrol, 0.9 mg catechins, 5.8 mg cyanidin, 6.7 mg malvidin, 1.5 mg quercetin, 0.3 mg kaempferol, 0.3 mg isorhamnetin | 42 | Day prior | GXT - Individualized start speed and 0% incline. Grade increased by 2.5% every 2 mins (walk/run) | → TTE | - | → Max V̇O_2_, VE, HR, RER or RPE |  |
| Toscano et al. 2015 | DB, P | 28 (6); 39.8 ± 8.5; 43.2 ± 8.5; Recreationally active runners | Grape, 10 ml/kg Brazilian purple grape juice | TPC - 1.82 g/L, 52.58 mg/L anthocyanins | 28 | 48 h | TTE at Anaerobic Threshold (running) | → TTE |  |  | ↑ TAC & Uric acid → MDA, LDH & CK |
| Deley et al. 2017 | DB, CO | 48 (0); 31.0 ± 6.0; V̇O_2max_ N/A; Physically active | Grape and apple, 500 mg capsules of grape and apple extract | TPC - 300 mg | 1 | 1 h | TTE at 70% PPO (cycling) | ↑ TTE |  | ↓ V̇O_2_ recovery speed → Mean or max HR, V̇O_2_, VE & HRR |  |
| Dean, Braakhuis & Paton 2009 | DB, CO | 8 (0); 36.4 ± 6.1; 52.5 ± 6.1; Moderately well-trained cyclists | Green tea, 270 mg GTE | 269.7 mg EGCG | 6 | 2 h | 40 km TT (cycling) | → TTC | - | ↑BLA at 40 mins of TT ↓ Fat oxidation at 50-60 mins of submax  → Submax BGL, fat oxidation at 25-mins; TT mean HR & BLa | MBIs |
| Eichenberger et al. 2010 | DB, CO | 9 (0); 32.2 ± 6.3; 58.1 ± 6.3; Trained cyclists | Green tea, 470 mL GTE beverage | 159 mg total catechins, 68 mg EGCG | 21 | 3 h | ~30-min TT (cycling) | → TTC | - | → Submax & TT HR, V̇O_2_, RER, RPE, BGL, FFA, & BLa |  |
| Kuo et al. 2014 | DB, P | 40 (0); 20 ± 1; 41.4 ± 4; Sedentary | Green tea, 250 mg GTE | 207 mg catechins, 121 mg EGCG | 28 | >48 h | GXT - "Modified" incremental treadmill test (running) | → TTE | - | → V̇O_2max_ | → MDA, TAC & CK |
|  |  |  |  |  |  |  |  | → TTE | - | → V̇O_2max_ | → MDA, TAC & CK |
| Martin et al. 2014 | DB, CO | 14 (0); 24 ± 3; 48 ± 7; Active in aerobic activities | Green tea, GTE (Dose N/A) | TPC - 1000 mg, 900 mg catechins, 450 mg EGCG | 2 | 4 h | 250 kJ TT (cycling) | → TTC | → SBP & DBP | ↑ Submax plasma glycerol ↓ Submax HR → Submax plasma lactate, V̇O_2_, CO_2_, fat oxidation or RER; TT mean HR |  |
| Abbey et al 2009 | SB, CO | 12 (0); 22.5 ± 10.4; 50.1 ± 15.2; Experienced soccer players | Honey, 1 g CHO/kg honey |  | 1 |  | GXT - Multistage 20 m shuttle test (running) | NA | - | - |  |
| Knab et al. 2014 | ?, P | 33 (9); 35.3 ± 8.1; 55.4 ± 6.5; Trained cyclists | Mixed fruit & vegetable juice, 6 tbsp freeze-dried juice powder | 696 mg GAE, | 17 | 1.25 h | 15-min TT (cycling) | → MPO | - | → Submax RPE or HR |  |
| Bell et al. 2014 | DB, P | 16 (0); 30 ± 8; 61.6 ± 10.4; Well-trained cyclists | Montmorency cherry, 60 mL juice | 547.02 mg anthocyanins | 5 | NR | TT - Multiple during 109-min simulated road race (cycling) | → Total work | - | - | → CK |
| Bell et al. 2015 | DB, P | 16 (0); 30 ± 8; 61.6 ± 10.4; Well-trained cyclists | Montmorency cherry, 60 mL juice | 547.02 mg Anthocyanins | 5 | NR | TT - Multiple during 109-min simulated road race (cycling) | → Total work | - | - |  |
| Clifford et al. 2013 | DB, CO | 9 (0); 32.1 ± 11.2; 52.4 (SD N/A); Moderately trained cyclists and triathletes | Montmorency cherry, 870 mg freeze-dried powder | 216 mg polyphenols | 3 | 2.5-3.5 h | 20 km TT (cycling) | → TTC | - | → Submax V̇O_2_, BLa, efficiency, HR or RER; TT HR & BLa |  |
| Keane et al. 2018 | DB, CO | 10 (0); 28 ± 7; 59.0 ± 7.0; Trained cyclists | Montmorency cherry, 60 mL concentrate | TPC - 178.75 mg, 73.5 mg anthocyanins | 1 | ~ 1.5 hours | TTE - 70% Δ (cycling) | → TTE | → NO_3_^-^, NO_2_^-^, DBP, MAP, AI & PMV ↓ SBP | → Submax & peak tissue oxygenation, BLa, & V̇O_2_ during _M_od & Sev-INT |  |
| Morgan, Barton & Bowtell 2019 | DB, CO | 8 (0); 19.7 ± 1.6; 62.3 ± 10.1; Trained, competitive cyclists | Montmorency cherry, 6 freeze-dried capsules | TPC - 462.8 mg; 256.8 mg anthocyanins | 7 | ~1.25 h | 15 km TT (cycling) | ↓ TTC | - | ↑ Submax BLa, resting tissue oxygenation → Submax tissue oxygenation, V̇O_2_ & RER; TT mean V̇O_2_, & end-BLA, RER & tissue oxygenation |  |
| Braakhuis, Hopkins & Lowe 2014 | SB, CO | 22 (22); 31 ± 8; 49 ± 4; Trained runners | New Zealand blackcurrant, 500 mL juice | 300 mg anthocyanins | 23 | NR | 5 km TT (running) | → TTC | - | - | N/A (MBIs) |
|  |  |  |  |  |  |  | GXT - 10 km race start speed, then increased by 1 km/h each min for 4 mins, then incline increased 1% each min (running) | → Peak velocity |  |  | N/A (MBIs) |
| Cook et al. 2015 | DB, CO | 14 (0); 38 ± 13; 53 ± 6; Trained cyclists | New Zealand blackcurrant, 1300 mg extract | 105 mg of anthocyanins | 7 | ~ 2.9h | 16.1 km TT (cycling) | ↓ TTC | → SBP & DBP | ↑ Post-TT BLa,   Fat oxidation at 65% V̇O_2max_ ↓RER at 65% V̇O_2max_  → Submax BLa, V̇O_2_, efficiency, HR, RER or fat oxidation; RER & Fat Oxidation at 45% & 55% V̇O_2max_ |  |
| Murphy, Cook & Willams 2017 | DB, CO | 10 (0); 30 ± 12; 55 ± 7; Trained cyclists | New Zealand blackcurrant, 300 mg extract | 105 mg anthocyanins | 7 | ~2.3 h | 4 km TT (cycling) | → TTC | - | → TT HR & or post-TT BLa & HR |  |
| Perkins et al. 2015 | DB, CO | 13 (0); 25 ± 4; 56 ± 4; Recreationally active in high-intensity, intermittent sports | New Zealand blackcurrant, 300 mg extract | 105 mg anthocyanins | 7 | 3.3 h | IGXT - Modified NIE intermittent test (running) | ↑ Total distance | - | → Submax HR, V̇O_2_, BLa & RPE during 1^st^ four IGXT stages; post-IGXT BLa |  |
| Potter et al. 2019 | DB, CO | 18 (0); 24 ±6; V̇O_2max_ N/A; Experienced climbers (bouldering and sport climbing) | New Zealand blackcurrant, 600 mg extract | 210 mg anthocyanins | 7 | ~2.5 h | TTE - Treadwall climb to exhaustion (climbing) | → TTE | - | → Peak or mean HR, mean RPE |  |
| Willems et al. 2015 | DB, CO | 13 (5); 38 ± 8; 49.1 ± 6.2; Triathletes | New Zealand blackcurrant, 6g powder | 138.6 mg anthocyanins | 7 | ~3.3 h | GXT - Workload increased by 30 W/min (cycling) | → PPO | → SBP, DBP & MAP ↓ TPR | ↑ Power at OBLA, BLa-Power curve ↓ BLa at PPO → Submax HR, SV & Q |  |
| Willems et al. 2016 | DB, CO | 13 (0); 22±1; 49.6±5.1; Recreationally active | New Zealand blackcurrant, 300mg extract | 106 mg of anthocyanins | 7 | ~4.5 h | ITTE - Loughborough Intermittent shuttle test part B (running) | → TTE | - | ↑Post-ITTE HR → Post-ITTE BLa. |  |
| Esquius et al. 2019 | DB, CO | 7 (0); 32.2 ± 4.3; V̇O_2max_ N/A; National level runners | Olive oil, 25 mL extra virgin olive oil |  | 1 | 1 h | GXT - Initial speed of 6.5 Km/h. Slope increased 1% every 4 min until 20%, then speed increased 1 km/h every 4 min (incline walk/run) | → TTE | - | ↑ PETCO_2_ at AT  ↓ VE & PETO_2_ at AT  → V̇O_2_, FeO_2_, FeCO_2_ & RR at AT; Peak PETCO_2_, VE & PETO_2_ at AnT |  |
| Allen et al. 1998 | DB, P | 28 (8); 23.4 ± 3.2; 48.1 ± 9.6; Healthy | *Panax Ginseng*, 200mg of 7% Panax Ginseng |  | 21 |  | GXT - 50 W/min increases (cycling) | → TTE |  | - |  |
| Engels, Said & Wirth 1996 | DB, P | 19 (19); 26.3 ± 3.9; 33.9 ± 5.7; Healthy | *Panax Ginseng*, extract equivalent to 1000 mg *Panax ginseng* root |  | 56-60 days | NR | GXT - Started at 50 W, increased by 25 W every 3 mins (cycling) | → Total work | - | → Submax & max V̇O_2_, RER, VE & HR |  |
| Gelabert-Rebato et al. 2019 | DB, CO | 12 (0); 21.3 ± 2.1; 49.4 ± 8.2; Active | Peanut and mango leaf, 50 mg peanut husk extract, 140 mg mango leaf extract | 42.5 mg luteolin, 100 mg mangiferin; | 2 | NR | GXT - Started at power at Fat_max_, then increased 15 W every min (cycling); TTE at 70% W_max_ | → PPO (GXT) and TTE | → SBP & DBP | ↑ Efficiency → BLa, RPE & RER. |  |
|  |  |  |  |  | 15 |  |  | → PPO (GXT) and TTE | → SBP & DBP | → BLa, RPE, RER & efficiency |  |
|  |  |  | Peanut and mango leaf, 100 mg peanut husk extract, 420 mg mango leaf extract | 95 mg luteolin,  300 mg mangiferin | 2 |  |  | → PPO (GXT) and TTE | → SBP & DBP | → BLa, RPE, RER & efficiency |  |
|  |  |  |  |  | 15 |  |  | → PPO (GXT) and TTE | → SBP & DBP | → BLa, RPE, RER & efficiency |  |
| Crum, Barnes & Stannard 2018 | DB, CO | 8 (2); 37 ± 11; 54.7 ± 7.2; Trained cyclists | Pomegranate, 15 mg/kg (815–1,350 mg) extract | TPC - 652–1,080 mg/day, 622.7-1031.4 mg ellagitannins, 89–147 mg NO_3_^-^ | 8 | 2.5 h | 5-min TT (cycling) | → MPO | ↑ NO_3_^-^ | ↓ Submax V̇O_2_ → No change in HR, V̇CO_2_or SmO2 or THb |  |
| Torregrosa-García et al. 2019 | DB, CO | 26 (0); 34.9 ± 10.0; 54.4 ± 9.0; Trained cyclists | Pomegranate, 750 mg extract | 225 mg α + β punicalagins | 15 | >5 h | GXT - Started at 60% V̇O_2max_, then increased 35 W every 3 mins (cycling) | ↑ TTE | - | ↑ Time to VT2 → Submax RPE, V̇O_2_ at VT2, V̇O_2max_ & post-TTE BLa | → CK |
| Trexler et al. 2014 | DB, CO | 19 (9); 22.2 ± 2.2; 51.3 ± 9.4; Highly active | Pomegranate, 1000 mg extract |  | 1 | 0.5 h | TTE - 90% V_max_ | ↑ TTE | ↑ Vessel diameter 30-mins post-Ex, blood flow 30 mins post-consumption → At exhaustion for both intensities |  |  |
|  |  |  |  |  |  |  | TTE - 100% V_max_ | ↑ TTE |  |  |  |
| Ueberschlag et al. 2016 | DB, P | 35 (16); 34 ± 7; V̇O_2max_ N/A; Competitive (≥'Local class') runners | Protandim, 675 mg: 225 mg milk thistle, 150 mg bacopa, 150 mg ashwagandha root, 75 mg turmeric, 75 mg green tea |  | 90 | NR | 5km TT (running) | → TTC | - | - | → GPx, SOD, TAC & TBARS |
| Kern, Heslin & Rezende 2007 | DB, CO | 8 (4); 29.7 ± 4.4; 56.4 ± 7.9; Trained cyclists, duathletes and triathletes | Raisins, 1 g CHO/kg California red raisins |  | 1 | 1.5 h | 15-min TT (cycling) | → Total work | - | → BGL & BLa. |  |
| Rietschier et al. 2011 | ?, CO | 10 (0); 24.4 ± 5.4; 52.3 ± 4.1; Trained cyclists & triathletes | Raisins, 168 g sun dried raisins |  | 1 | During  preload | 10 km TT (cycling) | → TTC | - | → Submax RPE, RER, efficiency, & CHO/fat utilisation |  |
| Bousetta et al. 2019 | SB, CO | 11 (?); 22.5 ± 0.5; 50.3 ± 0.1^a^; Experienced soccer players | Red Orange, 500 mL fresh red orange juice | 241 mg flavanones, 217.05 mg hesperidin, 24 mg of narirutin | 1 | 2.5 h | IGXT - Yo-Yo IR1 (running) | → Predicted V̇O_2max_ | → Pre- or post-Ex SBP & DBP | - | ↓ MDA & CK → TAC & LDH |
| Jówko et al. 2018 | DB, P | 26 (0); 20.7 ± 0.3; 49.5 ± 2.0; Active | *Rhodiola rosea*, 600 mg *Rhodiola rosea* extract | TPC - 141 mg | 28 | 1.5 h | IGXT - Started at 1W/kg, with increases of 0.75W/kg every 3 min after a 1-min rest period (cycling) | ↑ Peak power | - | ↓ Resting BLa → Power at LT; Max BLa, HR & V̇O_2_ | ↑ TAC → SOD & CK |
| Dowling et al. 1996 | DB, P | 20 (4); 37±8; 53.8 ± 5.9; Highly trained distance runners | Siberian ginseng, 3.2 mL |  | 14 | NR | GXT - Started at 6.4km/h and 8% incline. Speed increased 1.6km/h each minute (running) | → TTE | - | → Submax & max V̇O_2_, VE, RER, HR & RPE; Post-TTE or serum lactate |  |
|  |  |  |  |  | 28 |  |  | → TTE | - | → Submax & max V̇O_2_, VE, RER, HR & RPE; Post-TTE or serum lactate |  |
|  |  |  |  |  | 42 |  |  | → TTE | - | → Submax & max V̇O_2_, VE, RER, HR & RPE; Post-TTE or serum lactate |  |
| Eschbach et al. 2000 | DB, CO | 10 (0); 28±2; 57.3 ± 2.0; Cyclists (from local and regional cycling clubs) | Siberian ginseng, 1200 mg |  | 7 | NR | 10 km TT (cycling) | → TTC | - | → Submax plasma lactate, HR & V̇O_2_ |  |
| Kalafati et al. 2010 | DB, CO | 9 (0); 23.3 ± 1.7; 52.2 ± 1.8; Moderately trained runners | *Spirulina platensis*, 6 g |  | 28 | Day  prior | TTE - 95% V̇O_2max_ (running) | ↑ TTE | - | ↑ Preload fat oxidation  → Plasma volume | → Catalase, TAC, TBARS & CK |
| Wasuntarawat et al. 2010 | DB, CO | 17 (0); 21 ± 1.5; V̇O_2max_ N/A; Untrained | Thai ginseng, 1.35 g dried powder |  | 1 | 1.5 h | TTE - 65% PPO (cycling) | → TTE | - | → RPE & HR during & post-TTE |  |
| Areta et al. 2018 | DB, CO | 9 (0); 30 ± 3; 71 ± 6; Well-trained cyclists/triathletes | Yerba Mate, 5 g powder | TPC - 456 mg | 6 | 1.9 h | 30-min TT (cycling) | ↓ TTC | - | ↑ TT V̇O_2_; fat oxidation 30, 40 & 50% V̇O_2max_. → Fat oxidation at 60-90% V̇O_2max_ |  |

^1^ Unless otherwise indicated, measured at rest, and NO_2_^-^ and NO_3_^-^ are plasma-derived; ^a^ estimated V̇O_2max_ from maximal exercise test, ^b^ V̇O_2max_ assessed via hand cycling. Abbreviations - %Δ, power output at GET, plus given percentage of the difference between GET and V̇O_2max_; AnT, anaerobic threshold; AT, aerobic threshold; ATP, adenosine triphosphate; BGL, blood glucose levels; BLa, blood lactate; BUN, blood urea nitrogen; CHO, carbohydrate; CK, creatine kinase; CO, crossover; DB, double blind; DBP, diastolic blood pressure; EGCG, epigallocatechin gallate; FeCO_2_, expired fraction of carbon dioxide; FeO_2_, expired fraction of oxygen; FFA, free fatty acids; FMD, flow-mediated dilation; FRAP, ferric-reducing antioxidant power; g, gram(s); GAE, gallic acid equivalents; GET, gas exchange threshold; GPx, glutathione peroxidase; GTE, green tea extract; GXT, graded exercise test; h, hour(s); HbO_2_, oxyhemoglobin; HHb, deoxygenated hemoglobin; HR, heart rate; HRR, heart rate recovery; iEMG, integrated electromyography; IGXT, intermittent graded exercise test; IR1, intermittent recovery level one; ITT, intermittent time trial; ITTE, intermittent time to exhaustion; kg, kilogram(s); kJ, kilojoules; km, kilometre(s); LDH, lactate dehydrogenase; LT, lactate threshold; m, metre(s); MAP, mean arterial pressure; max, maximal; MBIs, magnitude based inferences; MDA, malondialdehyde ; mg, milligram(s); min, minute; mL, millilitre(s); mmol, millimoles; Mod-INT, moderate-intensity; MPO, mean power output; N/A, not available; NIRS, near-infrared spectroscopy; NO_2_^-^, nitrite; NO_3_^-^, nitrate; NOx, Nitric oxide; NR, not reported; O_2_, oxygen; OBLA, onset of blood lactate accumulation; ORAC, oxygen radical absorbance capacity; P, parallel; PE, physical education; PETCO_2_, end-tidal partial pressure of carbon dioxide; PETO_2_, end-tidal partial pressure of oxygen; PPO, peak power output; Q, cardiac output; RER, respiratory exchange ratio; RPE, rating of perceived exertion; RQ, respiratory quotient; RR, respiratory rate; SaO_2_, oxygen saturation; SB, single blind; SBP, systolic blood pressure; SD, standard deviation; Sev-INT, severe-intensity; SmO2, muscle oxygen saturation; SOD, superoxide dismutase; submax, submaximal; SV, stroke volume; TAC, total antioxidant capacity; TBARS, thiobarbituric acid reactive substances; TC, time constant; THb, Total hemoglobin; TPC, total phenolic content; TPR, total peripheral resistance; TT, time trial; TTC, time to complete; TTE, time to exhaustion; V̇CO_2_, volume of carbon dioxide; VE, ventilation; V_max_, maximal running velocity; V̇O_2_, volume of oxygen; V̇O_2max_, maximal volume of oxygen consumption; VT, (first) ventilatory threshold; VT2, second ventilatory threshold; W_max_, maximum aerobic power
