# Supplementary material for: Effect of food sources of nitrate, polyphenols, L-arginine and L-citrulline on endurance exercise performance: a systematic review and meta-analysis of randomised controlled trials
Source: J Int Soc Sports Nutr. 2021 Dec 29;18:76. doi: 10.1186/s12970-021-00472-y (PMC8715640; doi:10.1186/s12970-021-00472-y)
Supplement: Supplementary file 4 — Additional file 4. L-citrulline meta-analysis and sub-group analyses. Description: L-citrulline meta-analysis and sub-group analyses table. [file 12970_2021_472_MOESM4_ESM.docx]

Noah MA d’Unienville ^a,b^_,_ Henry T Blake ^a,b^_,_ Alison M Coates ^a,b^_,_ Alison M Hill ^b,c^_,_ Maximillian J Nelson ^a,b^ & Jonathan D Buckley ^a,b^, ‘Effect of food sources of nitrate, polyphenols, L-arginine and L-citrulline on endurance exercise performance: a systematic review and meta-analysis of randomised controlled trials’_,_ *Journal of the International Society of Sports Nutrition*

^a^ Allied Health and Human Performance, University of South Australia, Adelaide, Australia

^b^ Alliance for Research in Exercise, Nutrition and Activity (ARENA), University of South Australia, Adelaide, Australia

^c^ Clinical and Health Sciences, University of South Australia, Adelaide, Australia

Corresponding Author: Noah M. A. d’Unienville - Contact email: Noah.D'Unienville@unisa.edu.au

**Online Resource 4:** L-citrulline meta-analysis and sub-group analyses

|  | ***k*** | **n** | **SMD (95% CIs)** | **p** | **I^2^ (%)** |
| --- | --- | --- | --- | --- | --- |
| **Overall** | *4* | 50 | -0.03 [-0.09, 0.02] | 0.244 | 0 |
| **Blinding** |  |  |  |  |  |
| **Double blind** | 3 | 30 | 0.09 [-0.18, 0.35] | 0.546 | 0 |
| **Unclear** | 1 | 20 | -0.04 [-0.09, 0.02] | 0.189 | - |
| **Supplementation Length** | *Qb:* |  | *0.56* | *0.456* |  |
| **Acute** | 2 | 28 | 0.09 [-0.23, 0.4] | 0.607 | 0 |
| **Multiple days** | 2 | 22 | -0.04 [-0.09, 0.02] | 0.197 | 0 |
| **Sex** |  |  |  |  |  |
| **Male** | *3* | 39 | -0.03 [-0.09, 0.02] | 0.229 | 0 |
| **Female** | *1* | 11 | 0.03 [-0.41, 0.47] | 0.899 | - |
| **Performance Level** | *Qb:* |  | *0.56* | *0.456* |  |
| **PL1** | - |  |  |  |  |
| **PL2** | 2 | 28 | -0.04 [-0.09, 0.02] | 0.197 | 0 |
| **PL2 Females** | - |  |  |  |  |
| **PL2 Males** | 2 | 28 | -0.04 [-0.09, 0.02] | 0.197 | 0 |
| **TT** | 1 | 20 | -0.04 [-0.09, 0.02] | 0.189 | - |
| **TTE** | 1 | 8 | 0.08 [-0.44, 0.6] | 0.770 | - |
| **PL3** | 2 | 22 | 0.09 [-0.23, 0.4] | 0.607 | 0 |
| **PL3 Females** | 1 | 11 | 0.03 [-0.41, 0.47] | 0.899 | - |
| **PL3 Males** | 1 | 11 | 0.14 [-0.3, 0.58] | 0.548 | - |
| **GXT** | 1 | 11 | 0.14 [-0.3, 0.58] | 0.548 | - |
| **PL4** | - |  |  |  |  |
| **PL5** | - |  |  |  |  |
| **Exercise Mode** | *Q_b_:* |  | *0.56* | *0.456* |  |
| **Cycling** | 2 | 28 | -0.04 [-0.09, 0.02] | 0.188 | 0 |
| **Running** | 2 | 22 | 0.09 [-0.23, 0.4] | 0.607 | 0 |
| **Test Type** |  |  |  |  |  |
| **GXT** | 2 | 22 | 0.09 [-0.23, 0.4] | 0.189 | 0 |
| **TT** | 1 | 20 | -0.04 [-0.09, 0.02] | 0.770 | - |
| **TTE** | 1 | 8 | 0.08 [-0.44, 0.6] | 0.607 | - |
| **Test duration** |  |  |  |  |  |
| **5-10 mins (TTE)** | 1 | 8 | 0.08 [-0.44, 0.6] | 0.770 | - |
| **>60 mins (TT)** | 1 | 20 | -0.04 [-0.09, 0.02] | 0.189 | - |
| **Overall** | 2 | 28 | -0.04 [-0.09, 0.02] | 0.197 | 0 |

Note that all trials were of crossover design that reported l-citrulline content. Abbreviations – k, number of trials; n, pooled sample size; SMD, standardised mean difference (Hedge’s g); LCI, lower confidence interval; UCI, upper confidence interval; GXT, graded exercise test; TT, time-trial; TTE, time to exhaustion; PL performance level; Q_b_, between-group Q-statistic.
